# A Maternal System Initiating the Zygotic Developmental Program through Combinatorial Repression in the Ascidian Embryo

**DOI:** 10.1371/journal.pgen.1006045

**Published:** 2016-05-06

**Authors:** Izumi Oda-Ishii, Atsushi Kubo, Willi Kari, Nobuhiro Suzuki, Ute Rothbächer, Yutaka Satou

**Affiliations:** 1 Department of Zoology, Graduate School of Science, Kyoto University, Kita-Shirakawa Oiwake-cho, Sakyo, Kyoto, Japan; 2 Department of Evolution and Developmental Biology, Zoological Institute, University Innsbruck, Innsbruck, Austria; University of Oxford, UNITED KINGDOM

## Abstract

Maternal factors initiate the zygotic developmental program in animal embryos. In embryos of the chordate, *Ciona intestinalis*, three maternal factors—Gata.a, β-catenin, and Zic-r.a—are required to establish three domains of gene expression at the 16-cell stage; the animal hemisphere, vegetal hemisphere, and posterior vegetal domains. Here, we show how the maternal factors establish these domains. First, only β-catenin and its effector transcription factor, Tcf7, are required to establish the vegetal hemisphere domain. Second, genes specifically expressed in the posterior vegetal domain have additional repressive cis-elements that antagonize the activity of β-catenin/Tcf7. This antagonizing activity is suppressed by Zic-r.a, which is specifically localized in the posterior vegetal domain and binds to DNA indirectly through the interaction with Tcf7. Third, Gata.a directs specific gene expression in the animal hemisphere domain, because β-catenin/Tcf7 weakens the Gata.a-binding activity for target sites through a physical interaction in the vegetal cells. Thus, repressive regulation through protein-protein interactions among the maternal transcription factors is essential to establish the first distinct domains of gene expression in the chordate embryo.

## Author Summary

During animal development, transcription factors and signaling molecules transcriptionally regulate one another and constitute a gene regulatory network. This network is evoked by maternally provided factors. Many maternal factors are localized and thereby activate a set of genes in a specific region. In embryos of the chordate, *Ciona intestinalis*, three maternal factors with localized activities are known. The present study demonstrated that these localized maternal factors interact with one another through a fourth non-localized transcription factor, Tcf7, and negatively regulate one another. These repressive interactions are essential to establish the first distinct domains of gene expression and evoke the gene regulatory network properly. The findings indicate that not only activating target genes but also repressing activities of other transcription factors through protein-protein interactions are important to properly initiate the zygotic program. Intriguingly, in one repressive interaction, a transcription factor loses its binding activity for target sites through an interaction with another transcription factor. Thus, this study provides a description of the entire system in which maternal factors initiate the zygotic developmental program of the *Ciona* embryo.

## Introduction

In animal embryos, maternal information initiates the zygotic developmental program. Maternal factors are often specifically localized to set up pre-patterns. This mechanism has been extensively studied in embryos of invertebrates including sea urchin and flies [1, 2]. In syncytium embryos of *Drosophila*, maternally localized factors define embryonic axes and initiate specific gene expression patterns from the zygotic genome [1]. Several specifically localized maternal factors are also known in vertebrates [3–7]. However, the whole system by which localized maternal factors establish the initial zygotic gene expression patterns is not yet fully understood.

**Fig 1:**
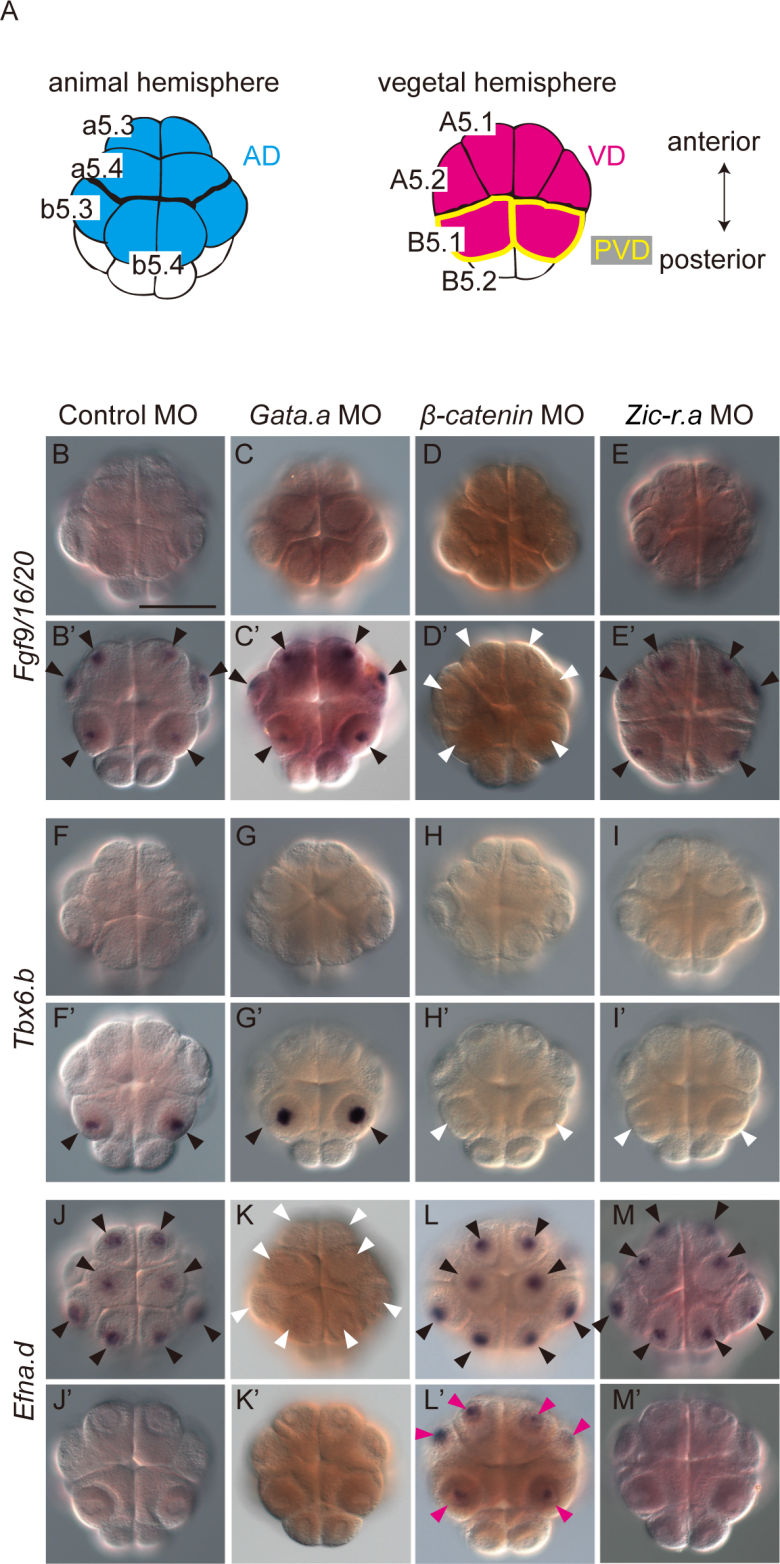
Three maternal factors regulate initial zygotic gene expression. (A) Schematics of the animal and vegetal hemispheres of the bilaterally symmetrical 16-cell embryo. Blastomere names are indicated in the left half of the bilaterally symmetrical embryo. Cell names in the animal hemisphere domain (AD; light blue) begin with a small letter. Cell names in the vegetal hemisphere domain (VD; magenta) begin with a capital letter. The posterior vegetal domain (PVD) consists of a pair of cells named B5.1, which are enclosed by yellow lines. (B–M) Expression of (B-E, B’-E’) *Fgf9/16/20*, (F–I, F’–I’) *Tbx6*.*b*, and (J–M, J’–M’) *Efna*.*d* in 16-cell embryos injected with (B, B’, F, F’, J, J’) a control MO, (C, C’, G, G’, K, K’) *Gata*.*a* MO, (D, D’, H, H’, L, L’) *β-catenin* MO, or (E, E’, I, I’, M, M’) *Zic-r*.*a* MO. White arrowheads indicate loss of expression. Magenta arrowheads indicate ectopic expression. (B–M) Animal views and (B’–M’) vegetal views are shown. Scale bar, 100 μm.

In the chordate, *Ciona intestinalis*, the first zygotic gene expression begins between the 8- and 16-cell stages. A comprehensive study has revealed that a limited number of regulatory genes encoding transcription factors and signaling ligands are expressed at the 16-cell stage [8–10]. The majority of genes activated at the 16-cell stage are expressed either in the entire animal hemisphere domain (AD), entire vegetal hemisphere domain (VD), or the posterior vegetal domain within the VD (PVD) (Figs 1A and S1). Cells in the AD mainly give rise to epidermal and neural cells, while cells in the VD give rise to mesendodermal tissues and the nerve cord. Muscle and mesenchymal cells are mainly derived from cells in the PVD.

Three maternal factors, β-catenin, Gata.a, and Zic-r.a (also called Macho-1) are involved in establishing these initial gene expression patterns [11–15]. β-catenin and Gata.a are required for expression in the vegetal and animal blastomeres, respectively [11–13]. The activity of β-catenin is restricted to the VD [11, 16], where it is suggested to suppress Gata.a activity [13]. Zic-r.a is localized in the posterior-most cells and required for activation of genes in the PVD [14, 15, 17]. In addition to these three maternal factors, Pem-1 is also known to be localized in the posterior-most cells. In the present study, we did not consider the posterior-most vegetal cells or Pem-1, because Pem-1 is thought to maintain the posterior-most cells, from which germ cells are derived, in a transcriptionally quiescent state by suppressing RNA polymerase II functions [18, 19].

It is not yet known whether combinatorial regulation by the above three maternal factors, β-catenin, Gata.a, and Zic-r.a, is sufficient to establish the initial zygotic gene expression pattern at the 16-cell stage. Furthermore, is it not understood how these maternal factors interact with each other to define precise regions of gene expression. Here, we describe the whole system that initiates the zygotic developmental program.

## Results

### Maternal factors initiating the zygotic genetic program

To understand how maternally expressed β-catenin, Gata.a, and Zic-r.a regulate gene expression at the 16-cell stage, we re-examined expression of genes that are activated at the 16-cell stage in morphant embryos of either of *β-catenin*, *Gata*.*a*, or *Zic-r*.*a*.

In normal embryos, *Fgf9/16/20* is expressed in the VD, and the same expression pattern was observed in embryos injected with a control morpholino oligonucleotide (MO). The expression of *Fgf9/16/20* was lost in β-catenin morphants as a previous study has shown [20], whereas *Fgf9/16/20* expression was scarcely affected in morphants of *Gata*.*a* or *Zic-r*.*a* (Fig 1B–1E; S1 Table). These results suggest that *Fgf9/16/20* is regulated by β-catenin only. This finding is consistent with a previous study indicating similar regulation of *Foxd*.*b*, which is also expressed specifically in the VD [13, 21] (S2A–S2D Fig; S1 Table).

As observed in normal embryos, in embryos injected with the control MO, *Tbx6*.*b* was expressed only in the PVD. This expression was lost in *β-catenin* and *Zic-r*.*a* morphants, but it was unaffected in *Gata*.*a* morphants (Fig 1F–1I; S1 Table); thus, *β-catenin* and *Zic-r*.*a* coordinately activate *Tbx6*.*b* in the PVD.

In normal embryos and embryos injected with the control MO, *Efna*.*d* and *Tfap2-r*.*b* (AP-2-like2) are expressed in the AD. The expression of *Efna*.*d* and *Tfap2-r*.*b* was greatly reduced in *Gata*.*a* morphants, and expanded to the vegetal hemisphere in *β-catenin* morphants (Figs 1J–1M and S2E–S2H; S1 Table). This observation is consistent with a previous study in which a synthetic reporter construct with 12 Gata-binding sites behaved similarly [13]. On the other hand, *Efna*.*d* and *Tfap2-r*.*b* expression was unaffected in *Zic-r*.*a* morphants; thus, *Efna*.*d* and *Tfap2-r*.*b* are activated by Gata.a in the AD, and repressed by β-catenin in the VD.

**Fig 2:**
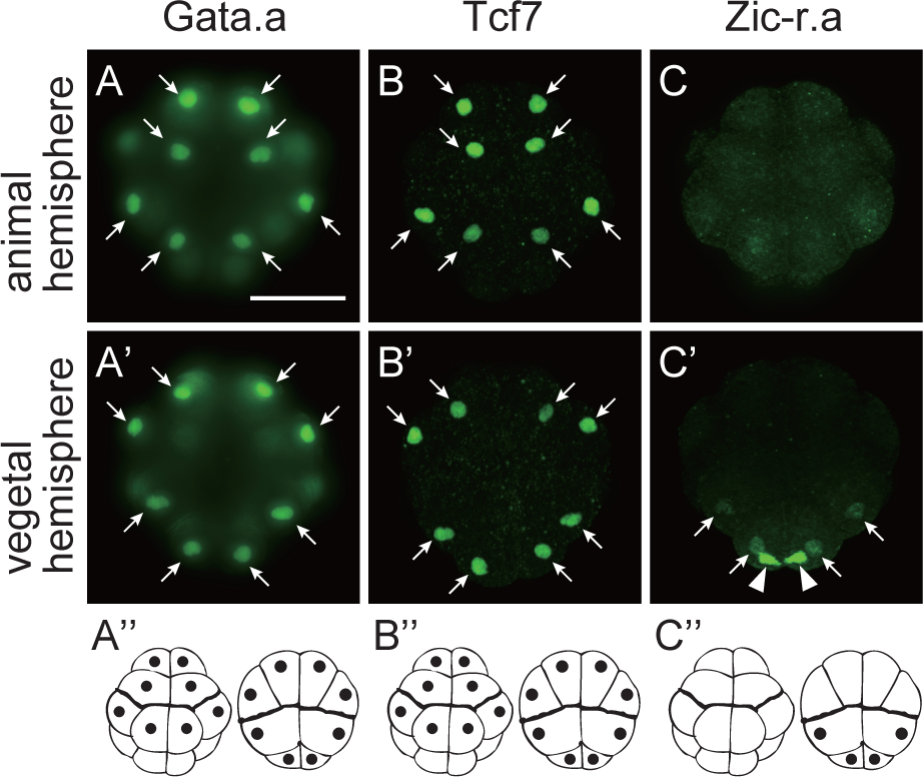
Distribution of Gata.a, Tcf7, and Zic-r.a at the 16-cell stage. Immunostaining of (A, A’) Gata.a, (B, B’) Tcf7, and (C, C’) Zic-r.a with specific antibodies. (A–C) Animal views and (A’–C’) vegetal views are shown. Cell nuclei were stained with these antibodies (arrows). Areas with maternal *Zic-r*.*a* mRNA expression [14, 15] were stained with the anti-Zic-r.a antibody (arrowheads). Photographs are Z-projected image stacks overlaid in pseudocolor. Signals detected in cell nuclei are depicted by black dots in (A”–C”). Scale bar, 100 μm.

We examined the distribution of Gata.a, Zic-r.a, and Tcf7, which employs β-catenin as a co-factor, by immunostaining with specific antibodies. Previous studies have shown that *Gata*.*a*, *Zic-r*.*a*, and *Tcf7* mRNAs are expressed mainly in the endoderm, nervous system, and mesenchyme, respectively, at the tailbud stage [8, 15]. Indeed, cell nuclei of these tissues were stained specifically with the antibodies (S3 Fig). At the 16-cell stage, Gata.a and Tcf7 proteins were observed equally in all nuclei of both of the AD and VD, while Zic-r.a was observed in all nuclei of the PVD and in the most posterior region where *Zic-r*.*a* mRNA is localized (Fig 2).

The above analyses raised three points. The first is how β-catenin and Zic-r.a cooperate to activate *Tbx6*.*b* specifically in the PVD. The second related point is why *Tbx6*.*b* is not activated in the anterior vegetal cells by β-catenin. The third is how β-catenin represses Gata.a activity in the VD.

### Enhancers driving expression in the vegetal hemisphere

To better understand the regulation of *Fgf9/16/20* expression, we prepared reporter gene constructs in which upstream regulatory sequences were fused to the green fluorescent protein (*Gfp*) gene. The reporter constructs were introduced into fertilized eggs by electroporation, and their expression was examined by in situ hybridization.

**Fig 3:**
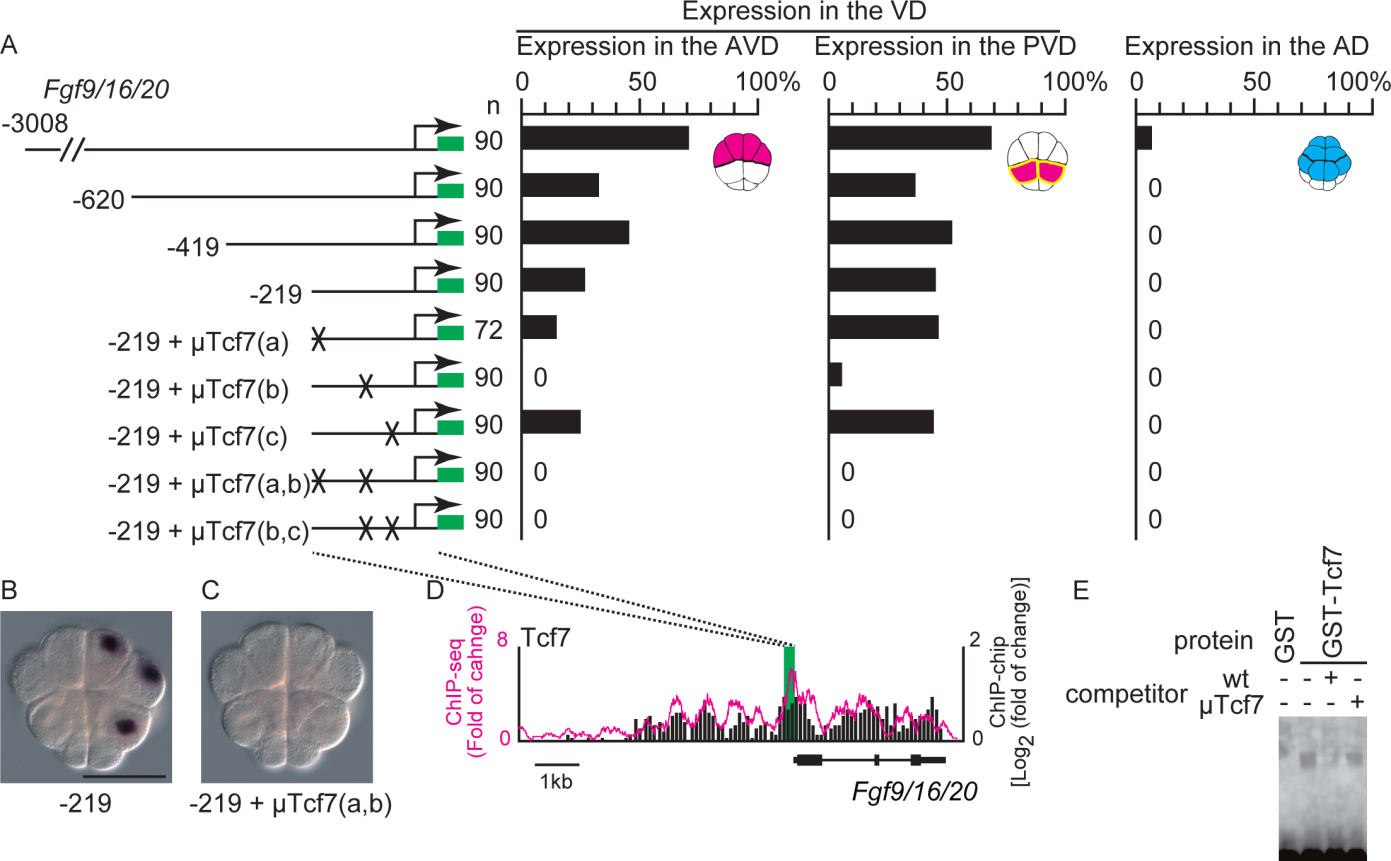
Tcf7-binding sites are critical for expression in the vegetal hemisphere domain (VD). (A) Analysis of a regulatory region of *Fgf9/16/20*. Illustrations on the left depict the constructs. Green boxes indicate the *Gfp* gene and SV40 polyadenylation signal. The numbers indicate the relative nucleotide positions from the transcription start site of *Fgf9/16/20*. Mutant Tcf7-binding sites are indicated by X. The graphs show the percentage of blastomeres expressing the reporter in the anterior vegetal blastomeres, posterior vegetal blastomeres, and animal blastomeres. Note that not all cells or embryos could express the reporter because of mosaic incorporation of the electroporated plasmid. (B, C) Images showing *Gfp* expression, which was revealed by in situ hybridization, in embryos electroporated with the fourth and eighth constructs shown in (A). Scale bar, 100 μm. (D) Mapping of the Tcf7 ChIP data onto genomic regions consisting of the exons and upstream region of *Fgf9/16/20*. ChIP-chip data are shown in bars. ChIP-seq data are shown as a magenta line. Each graph shows the fold enrichment (y-axis) for the chromosomal region over *Fgf9/16/20* (x-axis). A green box indicates the region essential for specific expression, which was revealed by reporter gene assays. This region overlapped peaks identified by the peak caller programs for ChIP-seq and ChIP-chip. (E) Gel-shift analysis showing that Tcf7-binding site b did not bind the GST protein but bound the Tcf7-GST fusion protein. The shifted band was greatly reduced by incubation with a specific competitor, but not a competitor with a mutant Tcf7-binding site b.

A series of deletion constructs showed that 219 bp upstream sequence of the transcription start site of *Fgf9/16/20* was sufficient to drive expression of the reporter gene specifically in the VD (Figs 3A, 3B and S4A). Because β-catenin functions as a cofactor of Tcf7, we searched for potential Tcf7-binding sites using a position weight matrix, and mutated the site with the highest score. The mutant sequence greatly reduced expression of the reporter [−219+μTcf(b)]. Although mutations introduced into two additional potential Tcf7-binding sites rarely affected expression of the reporter [−219+μTcf7(a) and −219+μTcf7(c)], double mutations of these sites in combination with the site of the highest score (site b) completely abolished reporter expression (Fig 3A and 3C). Therefore, the Tcf7 sites within the 219 bp region, especially site b, are responsible for the expression of the reporter.

We confirmed that Tcf7 bound to the 219 bp region by a chromatin immunoprecipitation (ChIP) assay using an antibody against *Ciona* Tcf7 followed by high-throughput sequencing (ChIP-seq) and microarray analysis (ChIP-Chip). As shown in Fig 3D, a clear ChIP peak was seen around the identified region. Indeed, peak caller programs for ChIP-seq and ChIP-chip data both identified peaks in this region (see Materials and Methods). A gel-shift assay also indicated that the TCF7-binding site of *Fgf9/16/20* bound Tcf7 specifically (Fig 3E).

We also analyzed upstream regions of *Foxd*.*b* and *Lefty*, which are expressed in the VD (S1 Fig). A region upstream of *Foxd*.*b* between −1241 and −1041 was required to drive expression of a reporter in the VD (S4B Fig). A previous study has shown that Tcf7 sites are essential for *Foxd* expression in a closely related species, *Ciona savignyi* [21]. We confirmed that mutations introduced into five putative Tcf7-binding sites in the upstream sequence of *Foxd*.*b* in *C*. *intestinalis* abolished reporter expression (S4B and S4C Fig). This region is highly conserved in the upstream region of a paralogous gene, *Foxd*.*a* (S4D Fig). This high conservation suggested the importance of this sequence. Although it prevented us from mapping ChIP data confidently to these regions, a gel-shift assay showed that the proximal Tcf7-binding site bound Tcf7 specifically (S4E Fig). We also confirmed that a 769 bp upstream region of *Lefty* was sufficient for expression in the VD (S4F Fig), and that this region contained a region that bound Tcf7 (S4G Fig). These observations are consistent with a previous study indicating that 12 repeats of the Tcf7-binding site can activate a reporter gene in the VD [13].

Our findings extended the results of previous studies [13, 21] for the following points. First, Tcf7 sites are essential for the specific expression of *Fgf9/16/20* and *Foxd*.*b*. Second, Tcf7 binds to the enhancers of genes that are specifically expressed in the VD. Thus, β-catenin and Tcf7 are the only factors required for specific expression in the entire VD.

### Tcf7-binding sites are required to drive expression in the posterior vegetal blastomeres

**Fig 4:**
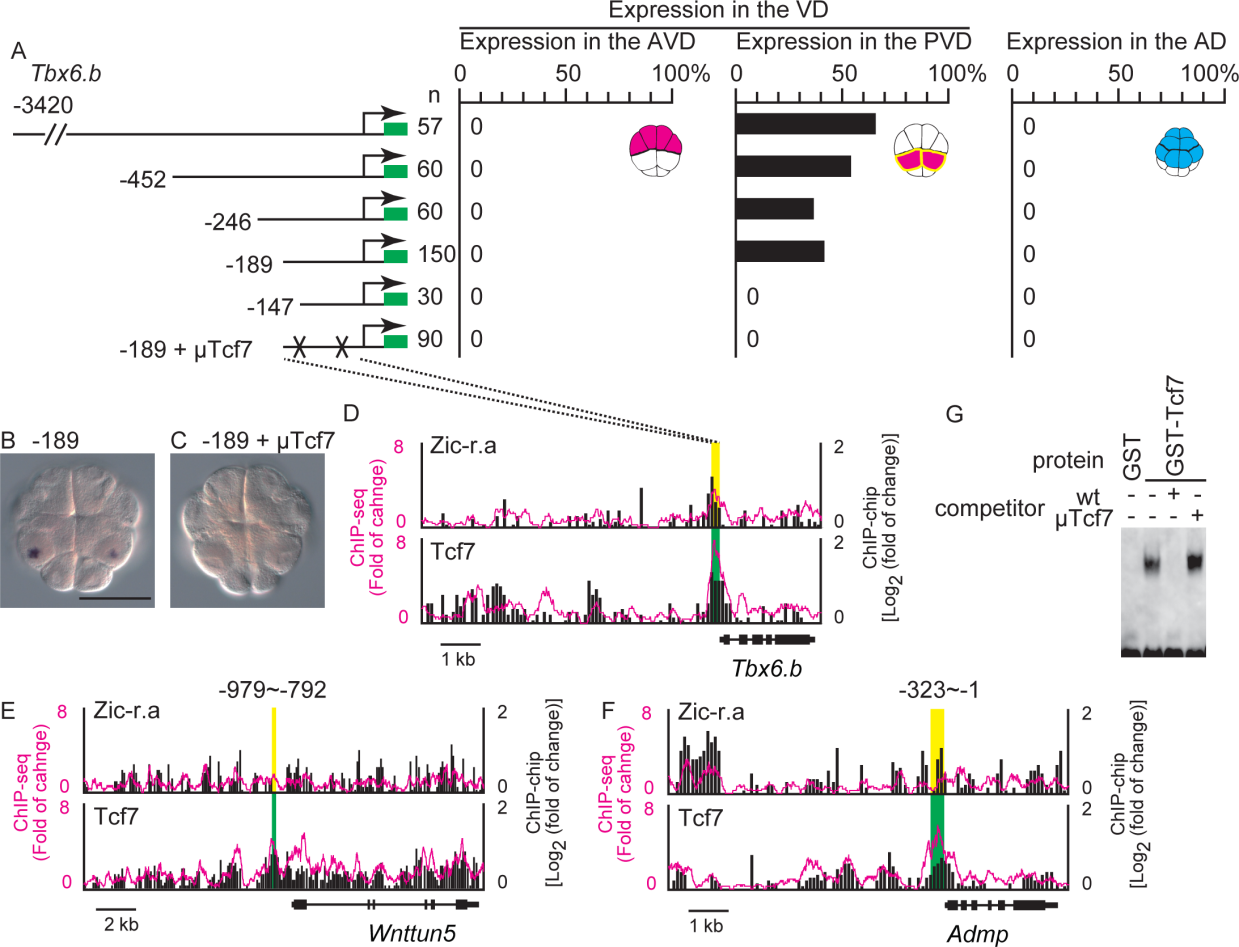
Tcf7-binding sites are critical for genes expressed specifically in the posterior vegetal hemisphere domain (PVD). (A–C) Analysis of a regulatory region of *Tbx6*.*b*. (A) Illustrations on the left depict the constructs. The numbers indicate the relative nucleotide positions from the transcription start site of *Tbx6*.*b*. Mutated Tcf7-binding sites are indicated by X. Graphs show the percentage of blastomeres expressing the reporter in the anterior vegetal blastomeres, in the posterior vegetal blastomeres, and in the animal blastomeres. Note that not all cells or embryos could express the reporter because of mosaic incorporation of the electroporated plasmid. (B, C) Images showing *Gfp* expression in embryos electroporated with the fourth and last constructs shown in (A). Scale bar, 100 μm. (D–F) Mapping of the Tcf7 and Zic-r.a ChIP data onto genomic regions consisting of the exons and upstream regions of (D) *Tbx6*.*b*, (E) *Wnttun5*, and (F) *Admp*. The ChIP-chip data are shown in bars, and the ChIP-seq data are shown as magenta lines. Each graph shows the fold enrichment (y-axis) for the chromosomal regions (x-axis). Green and yellow boxes indicate the regions essential for specific expression, which were revealed by the reporter gene assays shown in (A), and S5 Fig. Regions indicated by green boxes overlap peak regions identified by the peak caller programs for ChIP-seq and ChIP-chip, while the peak caller programs did not identify peaks within regions indicated by yellow boxes. (G) Gel-shift analysis showing that the proximal Tcf7-binding site did not bind GST protein but bound the Tcf7-GST fusion protein. The shifted band disappeared by incubation with a specific competitor, but not a competitor with a mutant Tcf7-binding site.

A previous study [22] showed that an upstream region between −862 and −2400 bp is required for the expression of *Tbx6*.*b* at the 32-cell stage. Our analyses using a series of deletion reporter constructs identified the −189 bp upstream of *Tbx6*.*b* as a region sufficient to drive reporter expression in the PVD at the 16-cell stage (Figs 4A, 4B and S5A). When two putative Tcf7-binding sites were mutated, the expression of the reporter was completely abolished (Fig 4A and 4C). ChIP showed that this region bound Tcf7 *in vivo* (Fig 4D), and a gel-shift assay showed that Tcf7 bound to the Tcf7 site *in vitro* (Fig 4G). In contrast, no significant peak regions for Zic-r.a were identified in this region by the peak caller programs, although a weak peak was visible (Fig 4D). We previously determined nucleotide sequences that preferentially bind Zic-r.a by an *in vitro* selection assay (see S11F Fig) [17]. However, we did not identify clear binding sites for Zic-r.a in the 189 bp region of *Tbx6*.*b* (S5A Fig). Therefore, the weak peak in this region might represent indirect binding of Zic-r.a (see below).

We also examined upstream regulatory sequences of *Wnttun5* and *Admp*, which are also expressed specifically in the PVD at the 16-cell stage (S1 Fig). A 188 bp region within the upstream sequence of *Wnttun5* (−792 to −979) was necessary for specific expression in the PVD (S5B and S5C Fig). Mutations introduced into either of two putative Tcf7-binding sites greatly reduced expression (S5B, S5D and S5E Fig), and this region bound Tcf7 *in vivo* and *in vitro* (Figs 4E and S5F). The 323 bp upstream region of *Admp* was sufficient to drive reporter expression specifically in the PVD at the 16-cell stage (S5G and S5H Fig), and this region bound Tcf7 *in vivo* (Fig 4F). The peak caller programs again did not identify peaks for Zic-r.a in these essential regions of *Wnttun5* and *Admp* (Fig 4E and 4F). Because Zic-r.a is required for the expression of *Wnttun5* and *Admp*, Zic-r.a might indirectly bind to the upstream regulatory regions of *Wnttun5* and *Admp* (see below).

### Zic-r.a physically interacts with Tcf7

**Fig 5:**
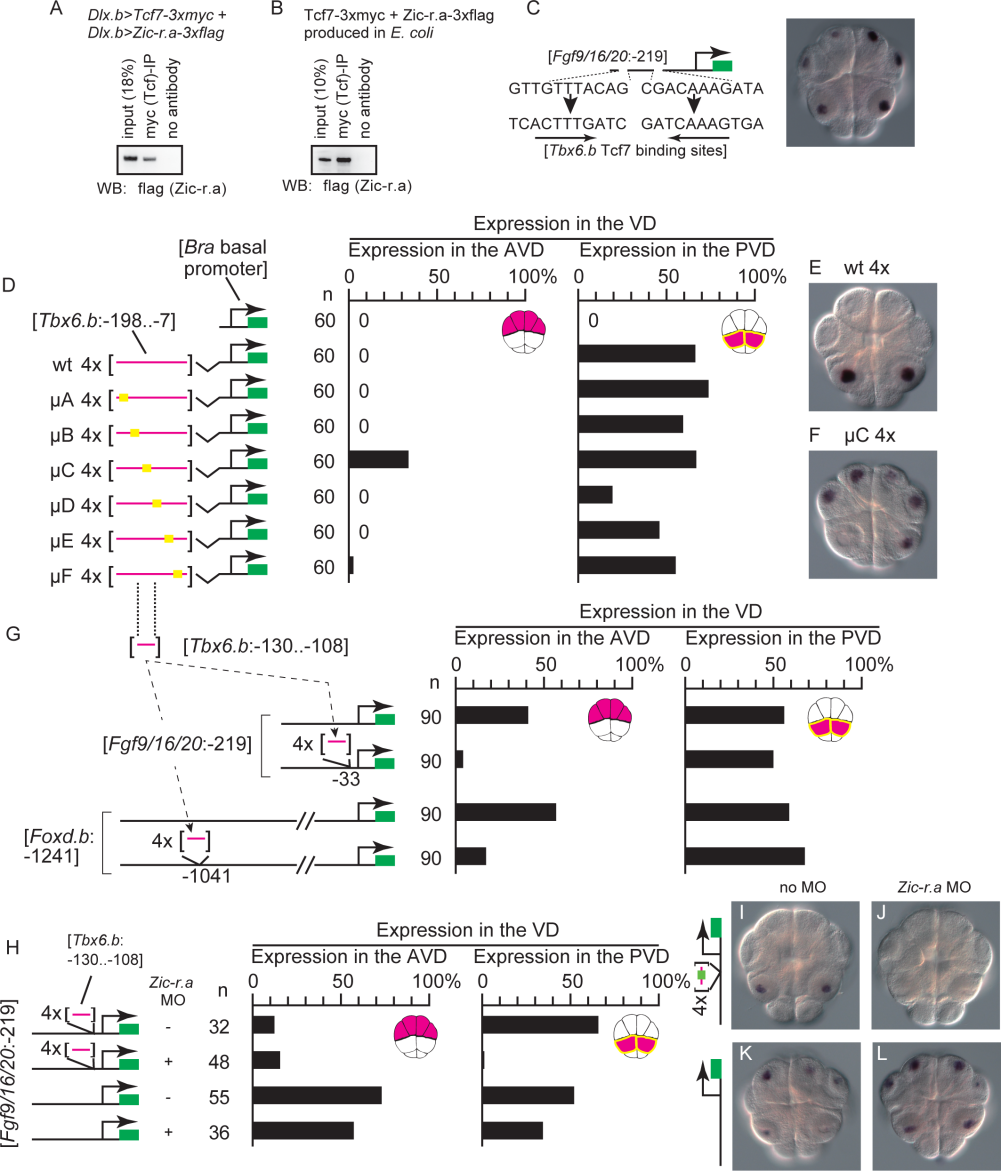
A repressive regulatory element required for specific expression of *Tbx6*.*b*. (A, B) Co-immunoprecipitation assays showing the interaction between Tcf7 and Zic-r.a. (A) 3xmyc-tagged Tcf7 and 3xflag-tagged Zic-r.a were misexpressed in epidermal cells using the *Dlx* upstream sequence, and a lysate of misexpressed embryos was used for the immunoprecipitation assay. (B) Recombinant 3xmyc-tagged Tcf7 and 3xflag-tagged Zic-r.a were produced in *E*.*coli*, and applied to the co-immunoprecipitation assay. (C) Replacing the Tcf7 sites critical for expression of *Fgf9/16/20* in the anterior and posterior vegetal cells with the proximal Tcf7 site critical for expression of *Tbx6*.*b* in the posterior vegetal cells did not affect specificity of the reporter gene expression. (D) A series of mutant constructs to identify the region required for specific expression in posterior vegetal cells is shown on the left. Four repeats of the critical upstream sequence of *Tbx6*.*b* with or without mutations were ligated to the *Brachyury* basal promoter. Graphs show the percentage of blastomeres expressing the reporter among the anterior vegetal blastomeres and among the posterior vegetal blastomeres. A graph showing the percentage in the animal hemisphere is not shown, because no embryos expressed the reporter in the animal hemisphere. Note that not all cells or embryos could express the reporter because of mosaic incorporation of the electroporated plasmid. (E, F) Images showing the reporter gene expression in embryos electroporated with the (E) second and (F) fifth constructs shown in (D). (G) Illustrations on the left depict the constructs. A potential repressive element in the upstream sequence of *Tbx6*.*b* was inserted into the upstream sequences of *Fgf9/16/20* and *Foxd*.*b*. Graphs on the right show the percentage of blastomeres expressing the reporter gene in the anterior vegetal blastomeres and in the posterior vegetal blastomeres. A graph showing the percentage in the animal hemisphere is not shown, because no embryos expressed the reporter in the animal hemisphere. (H–L) The repressive element of *Tbx6*.*b* directed specific expression in the posterior vegetal cells in a manner dependent on Zic-r.a activity. (H) Constructs depicted in the illustrations on the left were injected with or without an MO against *Zic-r*.*a*. Graphs on the right show the percentage of blastomeres expressing the reporter gene in the anterior vegetal blastomeres and in the posterior vegetal blastomeres. (I–L) Photographs of embryos injected with the *Fgf9/16/20* reporter construct with the repressive element of *Tbx6*.*b* (I, J) and the intact *Fgf9/16/20* reporter construct (K, L). The embryos shown in (J, L) were co-injected with the *Zic-r*.*a* MO.

Zic can function as a co-factor of transcription factors in vertebrates [23, 24]. Therefore, we hypothesized that Zic-r.a might function as a co-factor of Tcf7 and bind to the regulatory elements of *Tbx6*.*b*, *Wnttun5*, and *Admp* indirectly through Tcf7. To test whether Tcf7 interacted with Zic-r.a, 3xmyc-tagged Tcf7 and 3xflag-tagged Zic-r.a were overexpressed under the control of the upstream sequence of *Dlx*.*b* in epidermal cells of tailbud embryos [25]. By immunoprecipitation with a specific antibody against the myc-tag, we found that these two proteins can interacted with each other, although these two proteins might be expressed more abundantly in epidermal cells of experimental embryos than in the PVD of normal embryos (Fig 5A). We further confirmed this interaction *in vitro* using 3xmyc-tagged Tcf7 and 3xflag-tagged Zic-r.a proteins produced in *E*. *coli* (Fig 5B). Therefore, Zic-r.a may bind to DNA indirectly through binding to Tcf7, although our data do not rule out the possibility that Zic-r.a binds directly to regulatory elements of genes not tested in the present study.

### Zic-r.a is required to overcome repressive elements in the posterior vegetal cells

The above analyses showed that the Tcf7 sites in the upstream sequences of *Fgf9/16/20* and *Foxd*.*b* direct expression in the VD, and that the Tcf7 sites in the upstream sequences of *Tbx6*.*b* and *Wnttun5* do not direct expression in the anterior vegetal domain (AVD). To understand the differences between these two types of cis-regulatory elements, we first examined the possibility that Tcf7 sites in the upstream sequence of *Tbx6*.*b* and *Wnttun5* bound Tcf7 weakly, and that Zic-r.a enhanced activity of the β-catenin-Tcf7 complex in the PVD. We replaced two essential Tcf7-binding sites in the construct that contained the 219 bp upstream sequence of *Fgf9/16/20* (sites a and b; see Fig 3A) with the proximal Tcf7-binding site important for expression of *Tbx6*.*b*. This construct promoted reporter expression in the AVD and PVD (Fig 5C; n = 60, 57.1% of the anterior vegetal cells, 60.0% of the posterior vegetal cells, and no animal hemisphere cells expressed the reporter). Thus, the Tcf7-binding site in the upstream region of *Tbx6*.*b* was not likely to be qualitatively different from that of *Fgf9/16/20*.

Another possibility is that additional cis-regulatory elements are required for the different expression patterns. Because a construct consisting of twelve Tcf7-binding sites induces reporter expression in the VD [13], it is very unlikely that *Fgf9/16/20* and *Foxd*.*b* have additional cis-regulatory elements for expression in the AVD. Instead, it is more likely that *Tbx6*.*b* and *Wnttun5* have additional cis-regulatory elements that repress the activity of Tcf7-binding sites in the AVD. To investigate this possibility, we further narrowed down the cis-regulatory region of *Tbx6*.*b*. We first prepared a construct in which four repeats of a sequence that included the regulatory element of *Tbx6*.*b* were fused to the basal promoter of *Brachyury*. While the basal promoter alone cannot drive reporter expression [26], the fusion construct was specifically expressed in the PVD (Fig 5D–5F). Therefore, these sequences contained sufficient elements to drive the reporter expression in the PVD. Next, we prepared a series of *Tbx6*.*b* constructs in which various 15 bp regions were mutated. The mutant construct μC induced ectopic expression of the reporter in the AVD. The remaining constructs did not induce ectopic expression, although μD weakened reporter expression in the PVD. Therefore, the region mutated in μC (region C) is likely to repress expression in the AVD; note that our data do not rule out a possibility that there are additional repressive elements, because the mutations did not cover the entire region required for specific expression of *Tbx6*.*b*.

We next inserted four repeats of the 22 bp sequence containing region C into the downstream sequence of the critical Tcf7-binding site of the construct containing the 219 bp upstream sequence of *Fgf9/16/20* that was used in Fig 3. With this insertion, the construct rarely drove reporter expression in the AVD (Fig 5G). The same DNA fragment also reduced the activity of the essential regulatory region of *Foxd*.*b* in the anterior vegetal cells, when it was inserted downstream of the essential regulatory region (Figs 5G, S6A and S6B). Although the result does not necessarily indicate that the 22 bp region is the only repressive element in the upstream region of *Tbx6*.*b*, it showed that this 22 bp region has a repressive activity.

Expression of the construct with four repeats of the regulatory element of *Tbx6*.*b* and the basal promoter of *Brachyury*, which was used in Fig 5D, was dependent on *Zic-r*.*a*, because concomitant injection of the MO against *Zic-r*.*a* abolished reporter expression. However, the μC construct was expressed in both the AVD and PVD, even in *Zic-r*.*a* morphants (S6C Fig). Similarly, concomitant injection of the MO against *Zic-r*.*a* reduced PVD-specific expression of the construct containing the 219 bp upstream sequence of *Fgf9/16/20* and four repeats of the repressive elements, but did not affect expression of the construct containing only the upstream sequence of *Fgf9/16/20* (Fig 5H–5L). Therefore, this repressive element functions in both the AVD and PVD, and its repressive activity is overcome by Zic-r.a in the PVD of normal embryos.

We examined whether *Wnttun5* also had a similar repressive element. We prepared constructs in which four repeats of the sequence that contained the regulatory element of *Wnttun5* were fused to the basal promoter of *Brachyury*. While a construct containing a 297 bp upstream sequence between −994 and −697 drove reporter expression specifically in the PVD, constructs containing shorter upstream sequences (−994 to −735, −994 to −793, −994 to −853, and −979 to −860) drove reporter expression in both the AVD and PVD (S6D Fig). Four repeats of the sequence containing this putative repressive region repressed the activity of the regulatory region of *Foxd*.*b* in the AVD when it was inserted downstream of the essential regulatory region (S6E and S6F Fig). Thus, similar to *Tbx6*.*b*, *Wnttun5* also has a cis-regulatory element that repressed the reporter expression in the AVD.

### Interactions among β-catenin, Tcf7 and Gata.a create differential gene expression patterns between the animal and vegetal hemispheres

**Fig 6:**
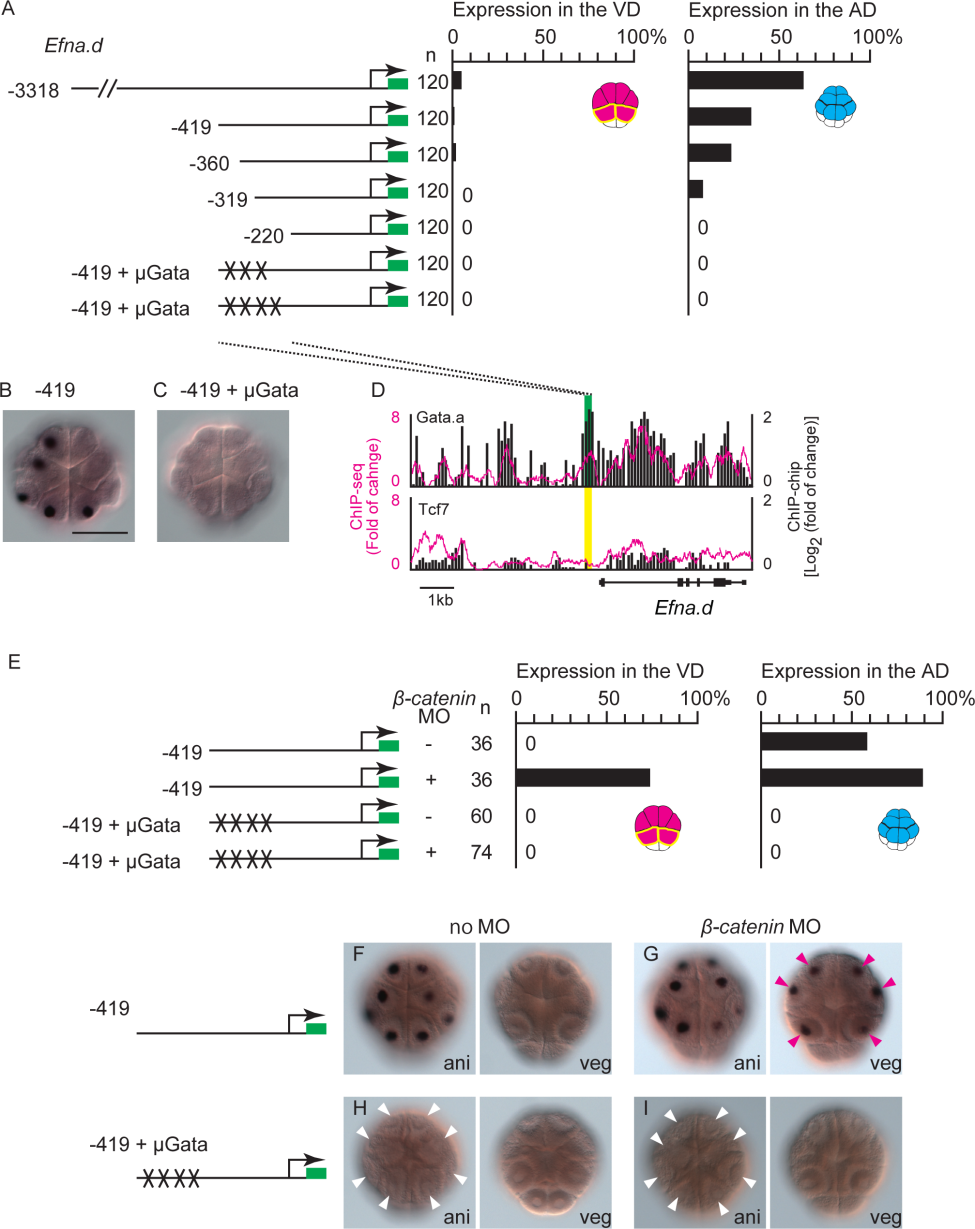
Gata-binding sites are critical for animal hemisphere-specific gene expression. (A) Analysis of a regulatory region of *Efna*.*d*. Illustrations on the left depict the constructs. The numbers indicate the relative nucleotide positions from the transcription start site of *Efna*.*d*. Mutant Tcf7-binding sites are indicated by X. Graphs show the percentage of blastomeres expressing the reporter in the vegetal blastomeres and in the animal blastomeres. Note that not all cells could express the reporter because of mosaic incorporation of the electroporated plasmid, and not all embryos could express the reporter either. (B, C) Images showing *Gfp* expression in embryos electroporated with the second and sixth constructs shown in (A). (D) Mapping of the Gata.a and Tcf7 ChIP data onto genomic regions consisting of the exons and upstream region of *Efna*.*d*. The ChIP-chip data are shown in bars. The ChIP-seq data are shown as a magenta line. Each graph shows the fold enrichment (y-axis) for the chromosomal regions (x-axis). Green and yellow boxes indicate the region essential for specific expression, which was revealed by the reporter gene assays. The green box indicates that a peak was identified by the peak caller programs for ChIP-seq and ChIP-chip in the region, while the yellow box indicates that no peaks were identified in the region. (E–I) Expression of *Gfp* in embryos injected with constructs containing the essential 419 bp region of *Efna*.*d*. Coinjection of the β-catenin MO evoked ectopic expression of the reporter with intact Gata-binding sites in the vegetal hemisphere, while it did not evoke expression of the reporter with mutant Gata-binding sites. (E) Graphs show the percentage of blastomeres expressing the reporter in the vegetal blastomeres and in the animal blastomeres. (F–I) Images showing *Gfp* expression in embryos injected with (F, G) the reporter construct with intact Gata sites or (H, I) mutant Gata sites. The embryos shown in (G, I) were co-injected with the *β-catenin* MO. Animal views (ani) and vegetal views (veg) are shown in each panel. White and magenta arrowheads indicate loss of expression and ectopic expression of the reporter, respectively. Scale bar, 100 μm.

Our previous study showed that specific expression of *Zfpm* (*Fog*) in the AD requires two Gata.a-binding sites [13]. Furthermore, a construct, in which twelve GATA-binding sites are placed in front of the *Brachyury* basal promoter (G12 construct), drives specific expression of a reporter in the AD [13]. In the present study, we also confirmed that Gata.a-binding sites of *Efna*.*d* were necessary for specific reporter expression in the AD. As shown in Fig 6A–6C, a series of deletion constructs of the upstream sequence of *Efna*.*d* revealed that the region between −419 and −220 was essential for driving reporter expression in the AD. Furthermore, mutations introduced into three or four putative Gata.a-binding sites within this region impaired the activity of this regulatory region. This finding was mostly consistent with a result published recently [27], except that a 319 bp upstream region, which lacked the critical Gata.a site identified in the previous study, activated the reporter weakly in our study. Indeed, ChIP analysis with an antibody against Gata.a showed that this region bound Gata.a *in vivo* (Fig 6D).

The 1513 bp upstream region of *Tfap2-r*.*b* (*AP-2-like2*) drove reporter expression specifically in the AD (S7B Fig), and a clear peak of Gata.a binding was observed within this region. Previous studies have shown that the 204, 975, and 314 bp upstream regions of *Gdf1/3-r*, *Fzd4*, and *Zfpm*, respectively, are sufficient to drive the expression of reporters in the AD [13, 27]. Clear peaks of Gata.a binding were observed within these upstream sequences (S7C–S7F Fig).

For further confirmation, we injected the *β-catenin* MO with the construct containing the 419 bp upstream region with intact or mutant Gata.a sites that were used in Fig 6A. While the reporter construct with the intact Gata.a sites was expressed ectopically in the VD of β-catenin morphants (Fig 6E, 6F and 6G), the reporter construct with mutant Gata sites was not expressed in the AD or VD (Fig 6E, 6H and 6I). Thus, ectopic expression of *Efna*.*d* in β-catenin morphants was dependent on these Gata.a sites.

**Fig 7:**
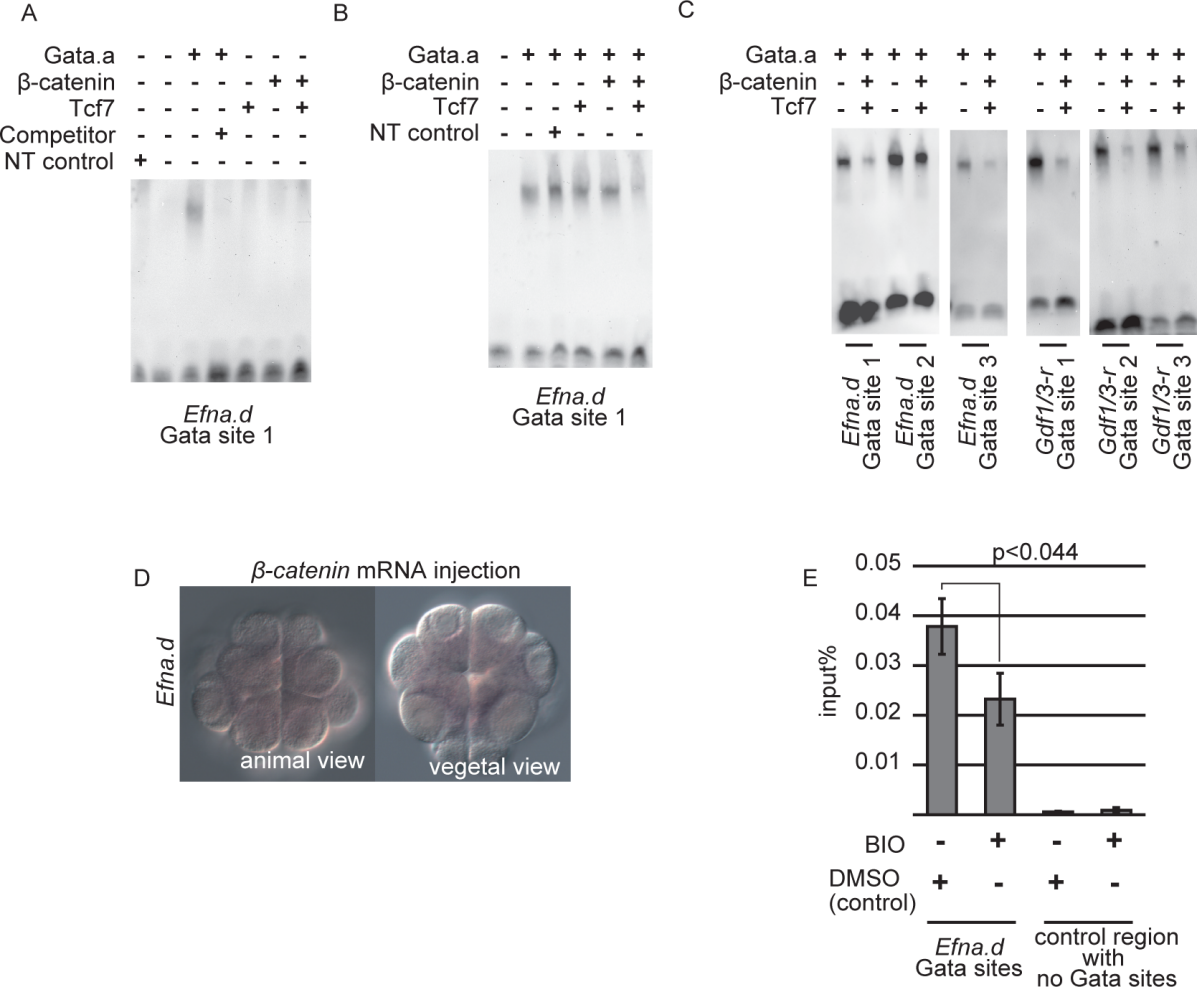
Gata.a binding activity is suppressed in a ternary complex with Tcf7 and β-catenin. (A) Gata.a protein produced *in vitro* specifically recognized the Gata site critical for *Efna*.*d* expression. As a negative control, we used a rabbit reticulocyte lysate that did not contain template plasmids (NT control). (B) Co-incubation of Gata.a with either β-catenin or Tcf7 did not affect the binding activity of Gata.a for the proximal Gata site (Gata site 1) in the upstream sequence of *Efna*.*d*, whereas co-incubation of Gata.a with β-catenin and Tcf7 reduced the binding activity of Gata.a. (C) Co-incubation of Gata.a with β-catenin and Tcf7 reduced the binding activity of Gata.a for Gata sites in the upstream sequences of *Efna*.*d* and *Gdf1/3-r*. (D) Expression of *Efna*.*d* was suppressed in embryos injected with β*-catenin* mRNA. (E) ChIP followed by quantitative PCR revealed that BIO treatment reduced Gata-a binding to the *Efna*.*d* upstream region. Error bars indicate standard errors of three independent experiments.

We next addressed how β-catenin suppresses *Efna*.*d* and *Tfap2-r*.*b* in the VD. The regulatory regions of *Efna*.*d*, *Tfap2-r*.*b*, *Gdf1/3-r*, *Fzd4*, and *Zfpm* did not bind Tcf7 (Figs 6D and S7C–S7F). This observation indicated that binding of the β-catenin and Tcf7 complex to the regulatory regions was not required for suppression of *Efna*.*d* expression in the VD. Hence, we reasoned that β-catenin might prevent Gata.a from binding to its target sites. To test this possibility, we performed gel-shift analysis. As shown in Fig 7A, Gata.a bound to the proximal site (site 1) of the three Gata sites tested in the sixth construct (in Fig 6A) of *Efna*.*d in vitro*, and the shifted band disappeared in the presence of a specific competitor. While this specific binding was not disrupted by co-incubation with either Tcf7 or β-catenin, it was reduced by co-incubation with both Tcf7 and β-catenin (Figs 7B and S8A). Co-incubation of Gata.a with β-catenin and Tcf7 also reduced Gata.a binding to other Gata sites in the upstream sequences of *Efna*.*d* and *Gdf1/3-r*, although different Gata sites showed different rates of reduction (Figs 7C and S8B). Therefore, in the VD of normal embryos, β-catenin and Tcf7 interact with Gata.a and suppress the binding activity of Gata.a.

For further confirmation, we used the 314 bp upstream sequence of *Zfpm*, which contains two critical Gata sites and is sufficient for activating a reporter in the AD (S9A Fig) [13]. When β-catenin was overexpressed in the AD under the control of the *Zfpm* enhancer, expression of a *LacZ* reporter under *Zfpm* was reduced markedly (S9B Fig). Similarly, injection of β*-catenin* mRNA greatly reduced the expression of *Efna*.*d* in the AD (Fig 7D; n = 75, 1.7% of anterior animal cells, 17% of posterior animal cells, and no vegetal hemisphere cells expressed *Efna*.*d*). In addition, treatment with BIO, which is a specific inhibitor of Gsk3 and thereby stabilizes β-catenin [16], reduced the expression (S9D and S9E Fig; n = 25, 0% of anterior animal cells, 0% of posterior animal cells, and no vegetal hemisphere cells expressed *Efna*.*d*). We confirmed that Gata-a binding to the *Efna*.*d* upstream region was significantly reduced in BIO-treated embryos by ChIP-qPCR (Fig 7E).

**Fig 8:**
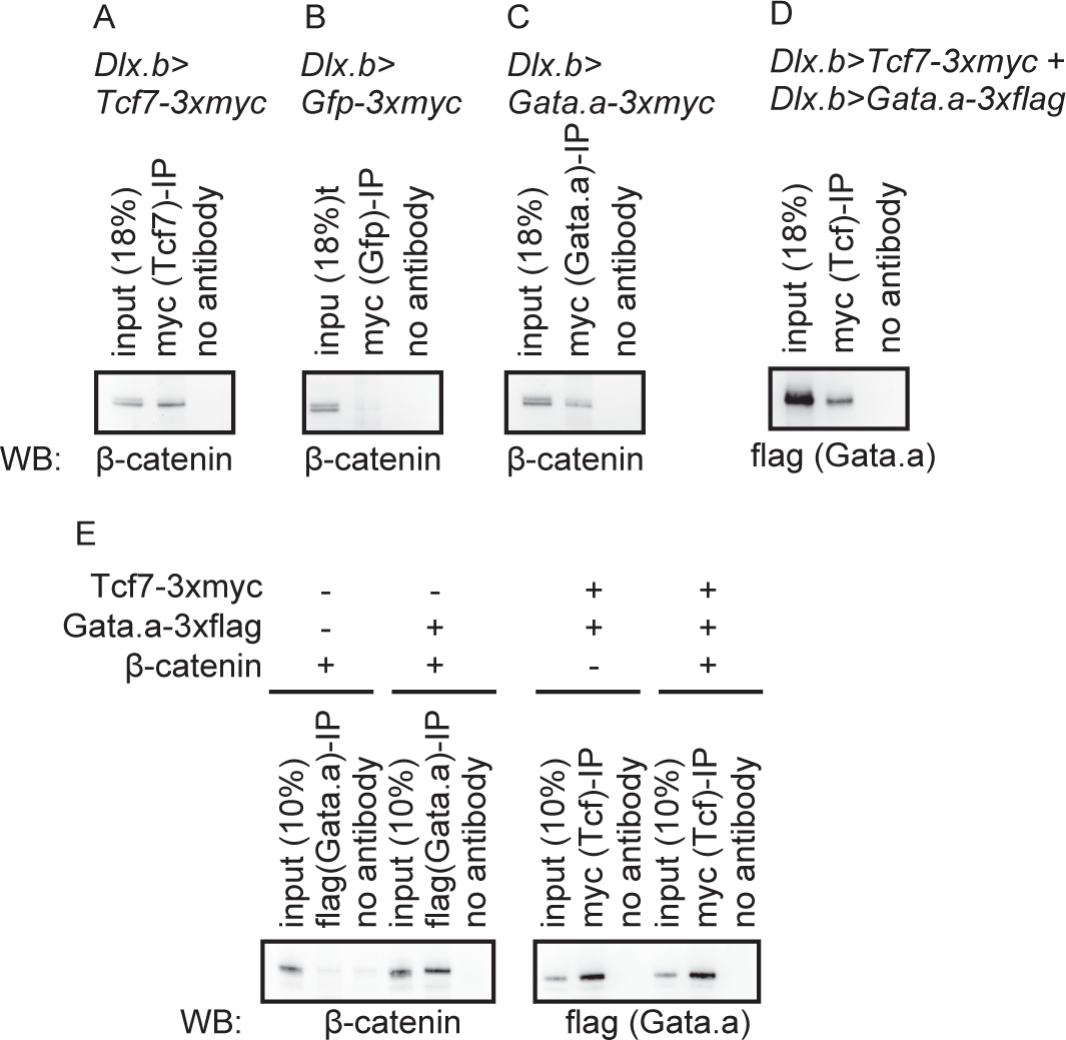
Interactions between Tcf7, Gata.a, and β-catenin. (A–D) Using the upstream sequence of *Dlx*.*b*, (A) 3xmyc-tagged Tcf7, (B) 3xmyc-tagged Gfp, (C) 3xmyc-tagged Gata.a, and (D) 3xmyc-tagged Tcf7 and 3xflag-tagged Gata.a were misexpressed in epidermal cells. Note that Gfp protein expressed using the upstream sequence of *Dlx*.*b* is present in both the nucleus and cytoplasm (S10 Fig). Lysates of embryos were used for immunoprecipitation assays with an anti-myc antibody. Western blotting was performed with an (A–C) anti-β-catenin antibody and (D) anti-flag antibody. (E) Immunoprecipitation assay to examine interactions among recombinant β-catenin, Tcf7, and Gata.a proteins produced in *E*. *coli*.

The above finding suggested that Gata.a physically interacted with β-catenin and Tcf7. To test this inference, we used embryos in which tagged proteins were misexpressed in the epidermis using the upstream sequence of *Dlx*.*b*. As expected, endogenously expressed β-catenin was co-immunoprecipitated with overexpressed myc-tagged Tcf7 but not myc-tagged Gfp (positive and negative controls, respectively; Fig 8A and 8B). We found that β-catenin was also co-immunoprecipitated with myc-tagged Gata.a (Fig 8C). In addition, flag-tagged Gata.a was co-immunoprecipitated with myc-tagged Tcf7 when they were co-expressed under the *Dlx*.*b* upstream sequence (Fig 8D). Although Tcf7 and Gata.a might be expressed more abundantly in epidermal cells of experimental embryos than in the AD of normal embryos, these results indicate that Gata.a can physically interact with Tcf7 and β-catenin *in vivo*. We also prepared recombinant proteins in *E*. *coli*, and confirmed that Gata.a interacted with β-catenin and Tcf7 (Fig 8E). The interaction between Tcf7 and Gata.a was not affected by the presence of β-catenin (Fig 8E). Thus, Gata.a can physically interact with Tcf7 and β-catenin.

## Discussion

**Fig 9:**
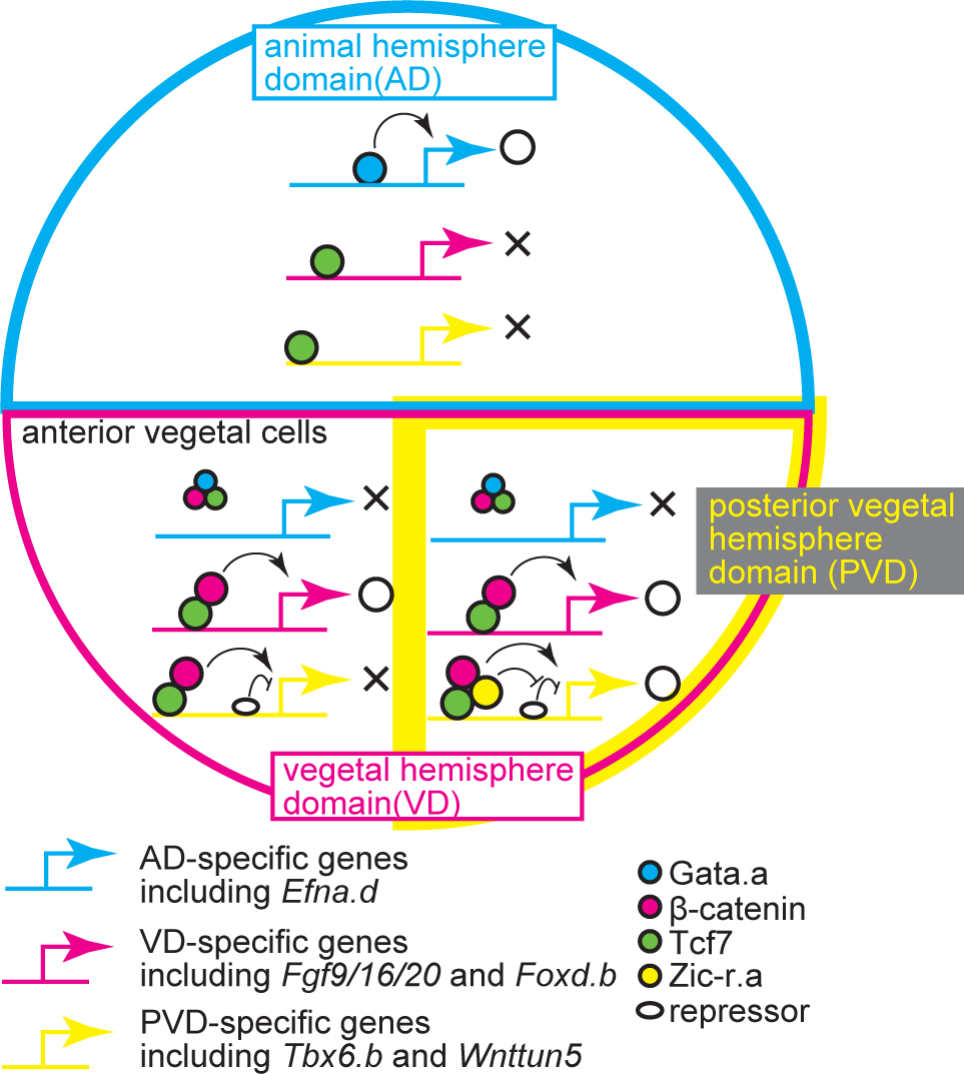
Regulatory system for the initial zygotic gene expression. Interactions among Gata.a, β-catenin, Tcf7, and Zic-r.a establish three distinct expression domains. See text for details.

We showed coordination of four maternal factors to activate the first zygotic gene expression (Fig 9). In *Ciona*, it is known that β-catenin activity is restricted to the VD. Therefore, β-catenin and Tcf7 activate gene expression in the VD. Because a complex of Gata.a, β-catenin, and Tcf7 interferes with Gata.a binding to Gata.a-binding sites, and the expression of *Efna*.*d* depends on Gata.a sites, *Efna*.*d* is not expressed in the VD. An interaction between TCF7L2 and GATA3 has been previously reported in human cell lines. This interaction is thought to tether TCF7L2 to the Gata3-binding site and repress transcription [28]. *Drosophila* Tcf can bind to sequences containing AGA[T/A]A[T/A] in addition to the canonical binding sequence [29]. However, such direct or indirect binding of Tcf7 to Gata sites was not detected by our ChIP analyses of the essential Gata sites of genes expressed in the animal hemisphere of *Ciona* embryos. Instead, in early *Ciona* embryos, formation of a complex of β-catenin, Tcf7 and Gata.a suppressed Gata.a-binding activity.

In *Xenopus*, maternal β-catenin establishes the dorsoventral axis [30, 31]. Gata-5 is expressed in early embryos under the control of maternal VegT and plays an important role in endoderm formation [32]. Hence, it is possible that the same mechanism operates in *Xenopus* embryos to establish clear boundaries of gene expression.

β-catenin also regulates *Tbx6*.*b* and *Wnttun5* that are specifically expressed in the PVD. *Tbx6*.*b* and *Wnttun5* have repressive cis-regulatory elements that prevent activation of these genes by β-catenin and Tcf7. Our data suggest that Zic-r.a can function in regulatory regions to overcome the repressive activity in the upstream sequences of *Tbx6*.*b* and *Wnttun5* without direct binding. First, within the essential regulatory region for specific expression of *Tbx6*.*b*, no clear Zic-r.a-binding sites were found. Second, we observed weak ChIP peaks for Zic-r.a, which were not identified as peaks by the peak finding programs, around the essential regulatory regions of *Tbx6*.*b* and *Admp*. Weak peaks may represent indirect binding of Zic-r.a, although ChIP peaks were hardly visible in the upstream sequence of *Wnttun5*. Third, Zic-r.a physically interacted with Tcf7, suggesting that Zic-r.a binds to the regulatory regions indirectly through Tcf7. Because Zic functions in *Xenopus* as a co-factor of the transcription factor Gli [23], and Zic forms a complex with Tcf4 in *Xenopus* and *Caenorhabditis* [33, 34], our finding is reasonable. Fourth, when the repressor elements were inserted, reporter constructs of *Fgf9/16/20* and *Foxd*.*b*, which are usually expressed independently of Zic-r.a, evoked *Zic-r*.*a*-dependent expression in the PVD. It is unlikely that the repressive elements bind Zic-r.a, because clear ChIP peaks were not found in this region. Nevertheless, we do not rule out the possibility that Zic-r.a binds directly to the regulatory regions of other genes expressed specifically in the PVD. Even if this were so, Zic-r.a would suppress repressor activity. Indeed, the ChIP assays of Zic-r.a identified more than 3800 peaks over the whole genome, and the Zic-r.a motif was enriched within these peaks, indicating that Zic-r.a binds directly to DNA as shown previously [17] (S11F Fig).

The evidence that *Tbx6*.*b* and *Wnttun5* have a repressive element is persuasive. The *Wnttun5* upstream sequence with the repressive activity contains several regions similar to the 15 bp repressive element of *Tbx6*.*b*, suggesting that a transcription factor commonly binds to these repressive elements.

A small number of genes are expressed at the 16-cell stage in patterns different from those of the genes examined in the present study. *Foxa*.*a* is expressed in the anterior animal cells and in the entire VD, *Soxb1* is expressed in the AD and in the AVD, and *Hes-a* is expressed in the AD and VD [8]. *Hes-a* might have two different enhancers that are responsible for expression in the AD and VD. However, the results of the present study cannot explain the expression patterns of *Foxa*.*a* and *Soxb1*. Thus, there may be additional maternal factors that regulate their expression.

In conclusion, we revealed a mechanism in which three maternal factors coordinate to establish three distinct expression domains, and this coordination is mediated through interactions with Tcf7. Our results show that negative regulatory mechanisms of the animal fate in the vegetal hemisphere and those of the posterior vegetal fate in the anterior vegetal hemisphere are important. Such negative regulatory mechanisms of developmental fates have not been well studied. However, they are likely to be important mechanisms of animal development.

## Materials and Methods

### Animals, whole-mount in situ hybridization, and gene identifiers

*C*. *intestinalis* (type A) adults were obtained from the National Bio-Resource Project for *Ciona*. cDNA clones were obtained from our EST clone collection [35]. Whole-mount in situ hybridization was performed as described previously [8].

Identifiers for genes examined in the present study were as follows: CG.KH2012.C8.396 for *Foxd*.*b*, CG.KH2012.C8.890 for *Foxd*.*a*, CG.KH2012.C2.125 for *Fgf9/16/20*, CG.KH2012.C3.716 for *Efna*.*d*, CG.KH2012.S654.3 for *Tbx6*.*b*, CG.KH2012.L20.1 for *Gata*.*a*, CG.KH2012.C1.727 for *Zic-r*.*a* (*Macho-1*), CG.KH2012.C9.53 for β*-catenin*, CG.KH2012.C6.71 for *Tcf7*, CG.KH2012.C9.257 for *Wnttun5*, CG.KH2012.C2.421 for *Admp*, CG.KH2012.C3.411 for *Lefty*, CG.KH2012.C4.547 for *Gdf1/3-r*, CG.KH2012.C7.43 for *Tfap2-r*.*b*, CG.KH2012.C6.162 for *Fzd4*, and CG.KH2012.C10.574 for *Zfpm* (*Fog*).

### Transcription factor site motifs

To identify potential Tcf7-binding sites, we used a position weight matrix available from the JASPAR database (MA0523) [36]. We calculated scores over candidate regulatory regions and excluded candidates without the core ‘TTT’ sequence. For Gata sites, we manually inspected ‘GATA’ sequences.

### Gene knockdown and overexpression

The MOs (Gene Tools, LLC) against *Gata*.*a*, β*-catenin*, and *Zic-r*.*a*, which block translation, have been used previously and their specificity evaluated [8, 12, 17]. We also used a standard control MO (5′-CCTCTTACCTCAGTTACAATTTATA-3′) purchased from Gene Tools, LLC. These MOs were introduced by microinjection under a microscope.

For synthetic mRNAs, coding sequences of *Gata*.*a*, *β-catenin*, and *Zic-r*.*a* were cloned into pBluscript RN3 [37], and synthetic mRNAs were transcribed using the mMESSAGE mMACHINE T3 Transcription Kit (Life technologies). β-catenin protein encoded by the synthetic mRNA lacked the N-terminal 45 amino acids that included phosphorylation sites for GSK3 [38]. Therefore, the overexpressed protein was detected in nuclei and expected to function in a constitutive active form (S12 Fig).

Reporter constructs were introduced into fertilized eggs by electroporation. When they were introduced with MOs or mRNAs, we used microinjection. Chromosomal positions of the upstream sequences for each series of reporter constructs are indicated in S4, S5 and S7 Figs. Deletions and mutations were introduced using the PrimeSTAR Mutagenesis Basal Kit (Takara). The mutated sequences are indicated in S4, S5 and S7 Figs. The *Brachyury* basal promoter consists of the chromosomal region, KhS1404:6203–6275. We randomly picked up experimental embryos, and scored the number of cells that expressed the reporter mRNA in all retrieved embryos. We did not quantify the reporter mRNA level.

All gene knockdown/overexpression experiments and reporter gene assays were performed at least twice with different batches of embryos.

### Antibody production and immunostaining

Coding sequences of *Gata*.*a*, *Tcf7*, and *Zic-r*.*a* were cloned into pET16b (Novagen), and His-tagged proteins were produced in *E*. *coli*. After purification with NiNTA resin (Qiagen), recombinant proteins were used for immunizing rabbits and polyclonal antibodies were obtained. These antibodies recognized Gata.a, Tcf7, and Zic-r.a as shown in S11A–S11C Fig, and the specific bands recognized by anti-Gata.a, -Tcf7, and -Zic-r.a antibodies were diminished by pre-incubation with their antigens but not with the control protein, GFP (S11D Fig).

To detect protein localization, embryos were fixed with 3.7% formaldehyde in PBS, treated with 3% H_2_O_2_ for 30 minutes, and then incubated with the antibodies in Can-Get-Signal-Immunostain Solution B (Toyobo). The signal was visualized with a TSA kit (Invitrogen) using horseradish peroxidase-conjugated goat anti-rabbit IgG and Alexa Fluor 488 tyramide. Control embryos incubated without primary antibodies yielded no signal.

### Chromatin immunoprecipitation

For ChIP assays, we collected 32-cell embryos from multiple batches. We used these multiple replicates for analysis, because a sufficient amount of material could not be obtained from a single batch. ChIP was performed as described previously [39]. Immunoprecipitated DNA was then split into two fractions. The first fraction was analyzed on a microarray as described previously [39] (GEO accession number: GSE70902). The second portion was subjected to sequence analysis using the Ion Plus Fragment Library Kit and a Ion PGM machine (SRA accession number: DRA003742). We obtained 1934656, 2225624, 3201484, and 4254462 tags for Gata.a ChIP, Zic-r.a ChIP, Tcf7 ChIP, and the whole cell extract control, respectively.

Analysis of the microarray data has been previously described [39]. In brief, peaks were called with two different programs independently [false discovery rate (FDR): 1%] [40, 41], and only peaks identified by both programs were adopted. Sequence data were analyzed with the program package Homer [42]. Using this package, immunoprecipitation efficiencies of Gata.a, Zic-r.a, and Tcf7 were estimated as 14%, 5%, and 16%, respectively. We confirmed that Gata, Tcf7, and Zic-r.a sites were successfully enriched in peaks identified in each immunoprecipitation using the mouse Gata-1 motif (MA0035.2 in the JASPAR database [37]), human TCF7L2 motif (MA0523.1 in the JASPAR database), and *Ciona* Zic-r.a motif [17], respectively (S11E–S11G Fig). Peaks were called without filtering based on local signals, because cis-regulatory elements are often densely encoded in the compact *Ciona* genome (FDR: <0.1%).

DNA immunoprecipitated with the anti-Gata.a antibody was subjected to quantitative PCR. An *Efna*.*d* upstream region (KHC3:2,807,067–2,807,176) was amplified with the following primers: 5′-CAATATTGCACACGGACACAAT-3′ and 5′-GGTCGCTGTTCGCTATCTCTC-3′. A control region (KHC13:910,574–910,630) was amplified with the following primers: 5′-TCCTTGTGCAACAAGTCGCT-3′ and 5′-GCGGCACGAGGTGTATGAA-3′.

### Co-immunoprecipitation assays

Gata.a, Zic-r.a, β-catenin, and Tcf7 with and without a 3×myc tag or 3×flag tag at their C-terminus were expressed in epidermal cells under the upstream sequence of *Dlx*.*b* as described previously [25]. β-catenin protein encoded by the *Dlx*.*b*>*β-catenin* construct lacked the N-terminal 45 amino acids that included phosphorylation sites for GSK3 [38]. Therefore, the overexpressed protein was expected to function in a constitutively active form. The lysates obtained from the resultant tailbud-stage embryos were used in co-immunoprecipitation assays with an anti-myc antibody (Abcam, ab9106) and the Dynabeads Co-Immunoprecipitation Kit (Life technologies). Immunoprecipitated samples were resolved on a sodium dodecyl sulfate-polyacrylamide gel and then subjected to western blot analysis with anti-FLAG (Sigma, F1840) and anti-β-catenin (a kind gift from Prof. Hiroki Nishida, Osaka University, Japan) antibodies. Signal detection was carried out using horseradish-peroxidase-labeled anti-mouse or rabbit IgG and an ECL-kit (GE Healthcare).

Recombinant Gata.a, Zic-r.a, β-catenin, and Tcf7 proteins with a 3×myc tag or 3×flag tag at their C-terminus were produced in *E*. *coli* using pET16b, and purified with NiNTA agarose (Qiagen). Co-immunoprecipitation assays and detection of immunoprecipitated samples were performed as described above.

### Gel-shift assays

Tcf7 protein for assays shown in Figs 3, 4, S4 and S5 was expressed as a GST-fusion protein in *E*. *coli* and purified with glutathione sepharose (GE healthcare). A double-stranded DNA for a Tcf7 site in the upstream sequence of *Fgf9/16/20* was prepared from the following oligonucleotides: 5′-AAAGTTCACCGACAAAGATAAGA-3′ and 5′-AAATCTTATCTTTGTCGGTGAAC-3′. The protruding ends were filled with biotin-11-dUTP (Thermo Fisher Scientific) using Taq DNA polymerase. Other probes were similarly prepared and their sequences are indicated in S4 and S5 Figs. Proteins and the biotin-labeled double-stranded DNA or unlabeled double-stranded DNA were mixed in 10 mM Tris (pH 7.5), 50 mM KCl, 1 mM DTT, 2.5 mM EDTA, 50 ng/μL poly(dAdT), 0.05% NP40, and 1 μg of the recombinant Tcf7-Gst fusion protein or GST protein. After incubation for 20 min, protein-DNA complexes were resolved by electrophoresis on a 6% native polyacrylamide gel in 0.5× TBE and then transferred to a nylon membrane. The Chemiluminescent Nucleic Acid Detection Module Kit (Thermo Fisher Scientific) was used to detect protein-DNA complexes.

Recombinant proteins for assays shown in Figs 7 and S8 were separately produced with a rabbit reticulocyte lysate system (TnT T7 Quick Coupled Transcription/Translation System, Promega). Probes were prepared using digoxigenin-11-dUTP (Roche). Their sequences are indicated in S7 Fig. Proteins and the digoxigenin-labeled double-stranded DNA or unlabeled double-stranded DNA were mixed in 10 mM Tris (pH 7.5), 50 mM KCl, 1 mM DTT, 5 mM MgCl_2_, 50 ng/μL poly(dIdC), and 0.05% NP40. The amount of proteins was determined empirically. Protein-DNA complexes were detected with an alkaline phosphatase-conjugated anti-digoxigenin antibody (Roche) and CDP-star (Roche). Bands were quantified as arbitrary units by a molecular imager (ChemiDoc XRS) using Quantity One software (Promega). Each experiment was independently performed two or three times.

## Supporting Information

S1 Table Expression of *Fgf9/16/20*, *Foxd*.*b*, *Tbx6*.*b*, *Efna*.*d*, and *Tfap2-r*.*b* in morphant embryos of maternal factors. Note that each embryo has four cells in each quadrant at the 16-cell stage. In the vegetal posterior quadrant, only the anterior B5.1 pair was counted, because the posterior B5.2 pair was not transcriptionally active.
(PDF)

S1 Fig Three major gene expression patterns at the 16-cell stage. Schematics of genes that are expressed (A) in the anterior and posterior vegetal cells, (B) in the posterior vegetal cells, and (C) in the animal hemisphere.
(PDF)

S2 Fig Expression of *Foxd*.*b* and *Tfap2-r*.*b* in morphants of the three maternal factors. Expression of (A–D, A’–D’) *Foxd*.*b* and (E–H, E’–H’) *Tfap2-r*.*b* in 16-cell embryos injected with (A, A’, E, E’) a control MO, (B, B’, F, F’) *Gata*.*a* MO, (C, C’, G, G’) *β-catenin* MO, or (D, D’, H, H’) *Zic-r*.*a* MO. White arrowheads indicate loss of expression, and magenta arrowheads indicate ectopic expression. (A–H) Animal views and (A’–H’) vegetal views are shown. Scale bar, 100 μm.
(PDF)

S3 Fig Distribution of Gata.a, Tcf7, and Zic-r.a at the tailbud stage. Immunostaining of (A) Gata.a, (B) Tcf7, and (C) Zic-r.a with specific antibodies. Images are Z-projected image stacks overlaid in pseudocolor. (A) *Gata*.*a* mRNA is expressed in endodermal cells at this stage [8], and Gata.a protein was detected in nuclei of endodermal cells. (B) *Tcf7* mRNA is expressed strongly in two cells of the brain [8], and Tcf7 protein was detected in nuclei of the two cells in the brain. (C) *Zic-r*.*a* mRNA is expressed widely in the nervous system [8, 15], and Zic-r.a protein was detected in nuclei of cells in the nervous system.
(PDF)

S4 Fig Regulatory elements of genes expressed specifically in the anterior and posterior vegetal hemisphere. (A) The upstream nucleotide sequence of *Fgf9/16/20* sufficient for driving reporter expression specifically in the vegetal hemisphere. Core sequences of the critical Tcf7-binding sites are shown in magenta, and the mutant sequences are shown below each of them in cyan. (B) Analysis of a regulatory region in *Foxd*.*b*. Illustrations on the left depict the constructs. Green boxes indicate the *Gfp* reporter gene and SV40 polyadenylation signal. The numbers indicate the relative nucleotide positions from the transcription start site of *Foxd*.*b*. Mutant Tcf7-binding sites are indicated by X. Graphs show the percentage of blastomeres expressing the reporter in the anterior vegetal blastomeres, in the posterior vegetal blastomeres, and in the animal blastomeres. (C) The upstream nucleotide sequence of *Foxd*.*b* required for driving reporter expression specifically in the anterior and posterior vegetal hemisphere. Core sequences of the critical Tcf7-binding sites are shown in magenta, and the mutant sequences are shown below each of them in cyan. (D) Alignment of the *Foxd*.*b* upstream sequence with the upstream sequence of its paralog, *Foxd*.*a*. (E) Gel-shift analysis showing that the proximal Tcf7 binding site in the upstream region of *Foxd*.*b* did not bind GST protein but bound the Tcf7-GST fusion protein. The shifted band disappeared by incubation with a specific competitor, but not a competitor with a mutant Tcf7-binding site. (F) The 769 bp upstream sequence of *Lefty* was sufficient for specific expression in the vegetal hemisphere. (G) Mapping of the Tcf7 ChIP data onto a genomic region consisting of the exons and upstream region of *Lefty*. The ChIP-chip data are shown in bars and the ChIP-seq data are shown as a magenta line. Each graph shows the fold enrichment (y-axis) for the chromosomal regions (x-axis). A green box indicates the essential upstream sequence of *Lefty* shown in (F). This region overlapped peaks identified by the peak caller programs for ChIP-seq and ChIP-chip. Nucleotide sequences enclosed by boxes in (A) and (C) were used for gel-shift assays.
(PDF)

S5 Fig Regulatory elements of genes expressed specifically in the posterior vegetal cells. (A) The upstream nucleotide sequence of *Tbx6*.*b* sufficient for driving reporter expression specifically in the posterior vegetal cells. Core sequences of the critical Tcf7-binding sites are shown in magenta, and the mutant sequences are shown below each of them in cyan. The positions of mutations shown in Fig 5D are enclosed by black boxes, and the mutant sequences are also shown in cyan. (B) Analysis of a regulatory region of *Wnttun5*. Illustrations on the left depict the constructs. Green boxes indicate the *Gfp* reporter gene and SV40 polyadenylation signal. The numbers indicate the relative nucleotide positions from the transcription start site of *Wnttun5*. Mutant Tcf7-binding sites are indicated by X. Graphs show the percentage of blastomeres expressing the reporter in the anterior vegetal blastomeres, in the posterior vegetal blastomeres, and in the animal blastomeres. (C, D) Images showing expression of the reporter in embryos electroporated with (C) the third and (D) last constructs shown in (B). Scale bar, 100 μm. (E) The upstream nucleotide sequence of *Wnttun5* required for driving reporter expression specifically in the posterior vegetal hemisphere. Core sequences of the critical Tcf7-binding sites are shown in magenta, and the mutantd sequences are shown below each of them in cyan. (F) Gel-shift analysis showing that the distal Tcf7-binding site in the upstream region of *Wnttun5* did not bind GST protein but bound the Tcf7-GST fusion protein. The shifted band disappeared by incubation with a specific competitor, but not a competitor with a mutant Tcf7-binding site. (G) Analysis of a regulatory region of *Admp*. Illustrations on the left depict the constructs. The numbers indicate the relative nucleotide positions from the transcription start site of *Admp*. Graphs show the percentage of blastomeres expressing the reporter in the anterior vegetal blastomeres, in the posterior vegetal blastomeres, and in the animal blastomeres. (H) The upstream nucleotide sequence of *Admp* sufficient for driving reporter expression specifically in the posterior vegetal hemisphere. Nucleotide sequences enclosed by red boxes in (A) and (E) were used for gel-shift assays.
(PDF)

S6 Fig Repressive elements required for specific expression in the posterior vegetal cells. (A)While the reporter gene was expressed in the anterior and posterior vegetal blastomeres under the control of the 1241 bp upstream sequence of *Foxd*.*b*, (B) insertion of four repeats of the 22 bp sequence within the upstream region of *Tbx6*.*b* suppressed the expression in the anterior vegetal cells. Images are embryos expressing the third and fourth constructs shown in Fig 5G. (C) The repressive element of *Tbx6*.*b* directed specific expression in the posterior vegetal cells in a manner dependent on Zic-r.a activity. Constructs depicted in the illustrations on the left were injected with or without an MO against *Zic-r*.*a*. The green boxes indicate the *Gfp* reporter gene and SV40 polyadenylation signal. Graphs on the right show the percentage of blastomeres expressing the reporter gene in the anterior vegetal blastomeres and in the posterior vegetal blastomeres. (D) A series of deletion constructs using the *Brachyury* basal promoter revealed a repressive element in the upstream sequence of *Wnttun5*. Illustrations on the left depict the constructs. Graphs show the percentage of blastomeres expressing the reporter in the anterior vegetal blastomeres, and in the posterior vegetal blastomeres. (E) The repressive element, which was identified in (D), was inserted into −1041 of the upstream sequence of *Foxd*.*b*. The graphs indicate that this insertion made the expression of the reporter specific for the posterior vegetal cells. Because no expression in the animal hemisphere was observed with the constructs shown in (C), (D) and (E), graphs for expression in the animal hemisphere are omitted. (F) Image showing expression of the reporter with the second construct shown in (E).
(PDF)

S7 Fig Regulatory elements of genes expressed specifically in the animal hemisphere. (A) The upstream nucleotide sequence of *Efna*.*d* sufficient for driving reporter expression specifically in the animal hemisphere. Core sequences of the critical Gata-binding sites are shown in magenta, and the mutant sequences (Fig 6A) are shown below each of them in cyan. Nucleotide sequences enclosed by boxes were used for the gel-shift assays shown in Fig 7. (B) The 1513 bp upstream sequence of *Tfap2-r*.*b* drove reporter expression specifically in the animal hemisphere. The illustration on the left depicts the construct. The green boxes indicate the *Gfp* reporter gene and SV40 polyadenylation signal. Graphs show the percentage of blastomeres expressing the reporter in the anterior and posterior vegetal blastomeres, and in the animal blastomeres. (C–F) Mapping of the Gata.a and Tcf7 ChIP data onto genomic regions consisting of the exons and upstream regions of (C) *Tfap2-r*.*b*, (D) *Gdf1/3-r*, (E) *Fzd4*, and (F) *Zfpm*. The ChIP-chip data are shown in bars and the ChIP-seq data are shown as magenta lines. Each graph shows the fold enrichment (y-axis) for the chromosomal regions (x-axis). Green and yellow boxes indicate the regions sufficient for specific expression that were revealed by the reporter gene assays shown in (B) and previous studies [13, 27]. Regions indicated by green boxes overlap peak regions identified by the peak caller programs for ChIP-seq and ChIP-chip, while the peak caller programs did not identify peaks within regions indicated by yellow boxes. (G) The upstream nucleotide sequence of *Gdr1/3-r* sufficient for driving reporter expression specifically in the animal hemisphere. Core sequences of the critical Gata-binding sites are shown in magenta. Nucleotide sequences enclosed by boxes were used for gel-shift assays shown in Fig 7.
(PDF)

S8 Fig Quantification of relative band intensities in gel-shift assays. (A) Shifted bands in the gel-shift assay shown in Fig 7B were quantified, and the relative intensity of shifted bands against the band in lane 2 of Fig 7B is shown. (B) Shifted bands in the gel-shift assay shown in Fig 7C were quantified, and the relative intensity of shifted bands is shown. Note that the intensity of shifted bands in lanes with only Gata.a protein may be underestimated as in this case probes were not used in sufficient excess. Black lines indicate standard deviations of two independent experiments.
(PDF)

S9 Fig Overexpression of *β-catenin* downregulates genes that are specifically expressed in the animal hemisphere domain. (A–C) A *LacZ* reporter construct containing the 314 bp upstream sequence of *Zfpm* was introduced by electroporation together with (A) an *mCherry* overexpression construct and (B) *β-catenin* overexpression construct. LacZ activity was detected at the gastrula stage using X-gal. (C) Stained embryos, in which 25% or more animal cells expressed the reporter, are shown. Error bars indicate standard deviations of two independent experiments. (D, E) Expression of *Efna*.*d* was suppressed in embryos incubated in sea water (D) without or (E) with BIO. Scale bar, 100 μm.
(PDF)

S10 Fig Localization of GFP. GFP, which was expressed using the *Dlx*.*b* upstream sequence, was observed in the nuclei and cytoplasm of a tailbud embryo. Scale bar, 100 μm.
(PDF)

S11 Fig Confirmation of the specificity of antibodies used for ChIP assays, and specific enrichment of the binding motifs in each ChIP-seq assay. (A–C) Western blot analyses showed that Tcf7, Zic-r.a, and Gata.a were specifically recognized by the antibodies used in the present study. A lysate of unfertilized eggs was used for all experiments. (D) Pre-adsorption tests to confirm antibody specificity. Specific bands recognized by anti-Gata.a, -Tcf7, and -Zic-r.a antibodies were diminished by pre-incubation with their antigens but not with GFP (S11D). (E–G) Enrichment of binding motifs of (E) human TCF7L2, (F) *Ciona* Zic-r.a and (G) mouse Gata1 shown on the right around peaks identified in ChIP-seq data using antibodies against Tcf7, Zic-r.a, and Gata.a.
(PDF)

S12 Fig β-catenin is expressed in nuclei of cells in the animal hemisphere by injection of synthetic *β-catenin* mRNA. (A) β-catenin was not detected in nuclei of cells in the animal hemisphere of a control embryo, while β-catenin associated with the cell membrane was detected readily. (B) β-catenin was clearly detected in nuclei of cells in the animal hemisphere of an embryo injected with β-catenin mRNA. High magnification views of regions enclosed by dashed-lines in the left panels are shown in the middle panels. Nuclei are indicated in the right panels by DAPI staining. Scale bars, 20 μm.
(PDF)
