# Correction: A Maternal System Initiating the Zygotic Developmental Program through Combinatorial Repression in the Ascidian Embryo

**DOI:** 10.1371/journal.pgen.1006392

**Published:** 2016-10-14

**Authors:** Izumi Oda-Ishii, Atsushi Kubo, Willi Kari, Nobuhiro Suzuki, Ute Rothbächer, Yutaka Satou

The number of embryos for the upper control reporter construct in panel E of S6 Fig is missing. This number is 90. Additionally, the percentages of expression of the lower reporter construct in panel E of S6 Fig are incorrect. The correct percentages are 5.8% in the AVD, and 61.6% in the PVD. Please view the correct S6 Fig here.

## Supporting Information

S6 Fig Repressive elements required for specific expression in the posterior vegetal cells. (A) While the reporter gene was expressed in the anterior and posterior vegetal blastomeres under the control of the 1241 bp upstream sequence of *Foxd*.*b*, (B) insertion of four repeats of the 22 bp sequence within the upstream region of *Tbx6*.*b* suppressed the expression in the anterior vegetal cells. Images are embryos expressing the third and fourth constructs shown in Fig 5G. (C) The repressive element of *Tbx6*.*b* directed specific expression in the posterior vegetal cells in a manner dependent on Zic-r.a activity. Constructs depicted in the illustrations on the left were injected with or without an MO against *Zic-r*.*a*. The green boxes indicate the *Gfp* reporter gene and SV40 polyadenylation signal. Graphs on the right show the percentage of blastomeres expressing the reporter gene in the anterior vegetal blastomeres and in the posterior vegetal blastomeres. (D) A series of deletion constructs using the *Brachyury* basal promoter revealed a repressive element in the upstream sequence of *Wnttun5*. Illustrations on the left depict the constructs. Graphs show the percentage of blastomeres expressing the reporter in the anterior vegetal blastomeres, and in the posterior vegetal blastomeres. (E) The repressive element, which was identified in (D), was inserted into −1041 of the upstream sequence of *Foxd*.*b*. The graphs indicate that this insertion made the expression of the reporter specific for the posterior vegetal cells. Because no expression in the animal hemisphere was observed with the constructs shown in (C), (D) and (E), graphs for expression in the animal hemisphere are omitted. (F) Image showing expression of the reporter with the second construct shown in (E).
(PDF)
