# Supplementary material for: A Maternal System Initiating the Zygotic Developmental Program through Combinatorial Repression in the Ascidian Embryo
Source: PLoS Genet. 2016 May 6;12(5):e1006045. doi: 10.1371/journal.pgen.1006045 (PMC4859511; doi:10.1371/journal.pgen.1006045)
Supplement: S1 Table — Note that each embryo has four cells in each quadrant at the 16-cell stage. In the vegetal posterior quadrant, only the anterior B5.1 pair was counted, because the posterior B5.2 pair was not transcriptionally active. (PDF) [file pgen.1006045.s001.pdf]

S1 Table. Expression of *Fgf9/16/20, Foxd.b, Tbx6.b, Efna.d,* and *Tfap2-r.b* in morphant embryos of maternal factors.

			Number of cells that expressed genes/Number of cells counted[*1]			
Genes examined	MO	Number of embryos	animal anterior (a-line)	animal posterior (b-line)	vegetal anterior (A-line)	vegetal posterior (B5.1)
Fgf9/16/20	not injected	173	0	0	640 / 692 (92.5%)	302 / 346 (87.3%)
	control standard MO	22	0	0	88 / 88 (100%)	42/ 44 (95%)
	β-catenin MO	59	0	0	0	0
	Gata.a MO	36	0	0	116 / 144 (81%)	48 / 72 (67%)
	Zic-r.a MO	29	0	0	93 / 116 (80%)	48 / 58 (83%)
Foxd.b	not injected	180	0	0	716 / 720 (99%)	353 / 360 (98%)
	control standard MO	31	0	0	124 / 124 (100%)	62 / 62 (100%)
	β-catenin MO	39	0	0	6 / 156 (3.8%)	4 / 78 (5.1%)
	Gata.a MO	33	0	0	128 / 132 (97%)	53 / 66 (80%)
	Zic-r.a MO	30	0	0	120 / 120 (100%)	59 / 60 (98%)
Tbx6.b	not injected	180	0	0	0	360 / 360 (100%)
	control standard MO	35	0	0	0	70 /70 (100%)
	β-catenin MO	25	0	0	0	0
	Gata.a MO	20	0	0	0	40 /40 (100%)
	Zic-r.a MO	30	0	0	0	2 / 60 (3.3%)

Efna.d	not injected	185	733 / 740 (99%)	737 / 740 (99%)	0	0
	control standard MO	35	140 / 140 (100%)	140/ 140 (100%)	0	0
	β-catenin MO	49	103 / 196 (53%)	165 / 196 (84%)	81 / 196 (41%)	22 / 98 (22%)
	Gata.a MO	55	21 / 220 (10%)	49 / 220 (22%)	0	0
	Zic-r.a MO	55	159 / 220 (72%)	193 / 220 (88%)	0	3 / 110 (2.7%)
Tfap2-r.b	not injected	139	490 / 556 (88%)	489 / 556 (88%)	0	0
	control standard MO	36	144 / 144 (100%)	144 / 144 (100%)	0	0
	β-catenin MO	27	98 / 108 (91%)	93 / 108 (86%)	100 / 108 (93%)	10 / 54 (19%)
	Gata.a MO	25	17 / 100 (17%)	15 / 100 (15%)	0	0
	Zic-r.a MO	30	107 / 120 (89%)	104 / 120 (87%)	0	0

[*1] Note that each embryo has four cells in each quadrant at the 16-cell stage. In the vegetal posterior quadrant, only the anterior B5.1 pair was counted, because the posterior B5.2 pair was not transcriptionally active.
