# Supplementary figures and images for: A Maternal System Initiating the Zygotic Developmental Program through Combinatorial Repression in the Ascidian Embryo
Source: PLoS Genet. 2016 May 6;12(5):e1006045. doi: 10.1371/journal.pgen.1006045 (PMC4859511; doi:10.1371/journal.pgen.1006045)

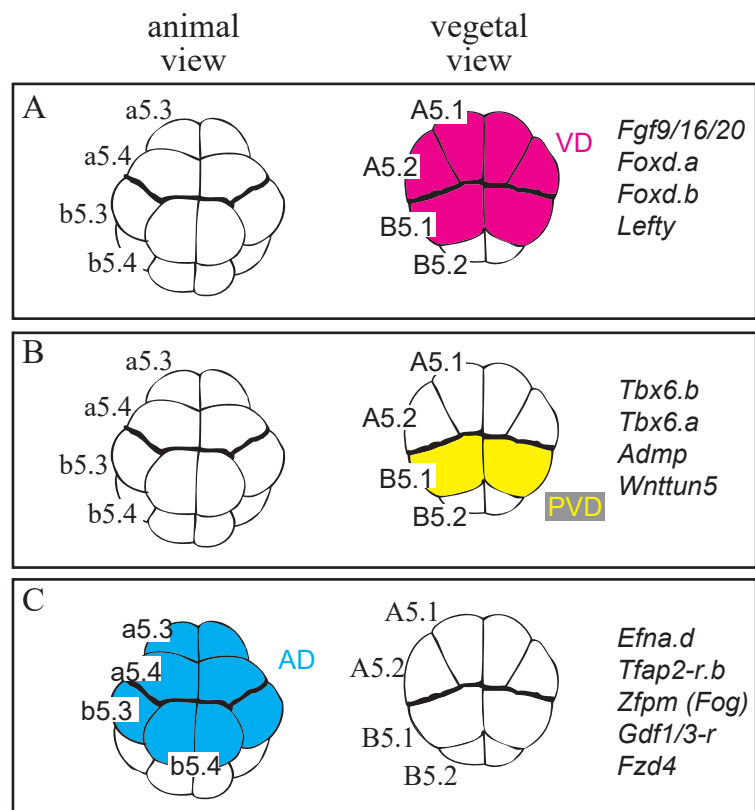

Supplement: S1 Fig — Schematics of genes that are expressed (A) in the anterior and posterior vegetal cells, (B) in the posterior vegetal cells, and (C) in the animal hemisphere. (PDF) [file pgen.1006045.s002.pdf]

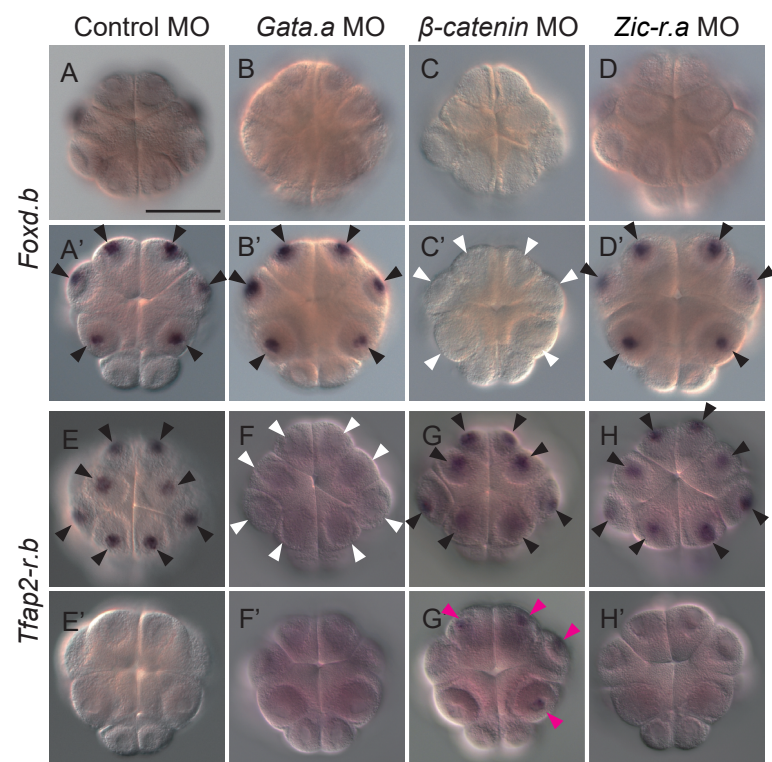

Supplement: S2 Fig — Expression of (A–D, A’–D’) Foxd.b and (E–H, E’–H’) Tfap2-r.b in 16-cell embryos injected with (A, A’, E, E’) a control MO, (B, B’, F, F’) Gata.a MO, (C, C’, G, G’) β-catenin MO, or (D, D’, H, H’) Zic-r.a MO. White arrowheads indicate loss of expression, and magenta arrowheads indicate ectopic expression. (A–H) Animal views and (A’–H’) vegetal views are shown. Scale bar, 100 μm. (PDF) [file pgen.1006045.s003.pdf]

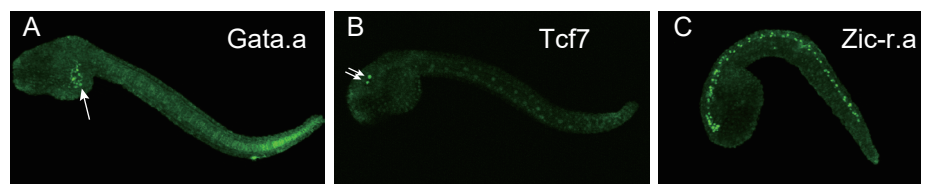

Supplement: S3 Fig — Immunostaining of (A) Gata.a, (B) Tcf7, and (C) Zic-r.a with specific antibodies. Images are Z-projected image stacks overlaid in pseudocolor. (A) Gata.a mRNA is expressed in endodermal cells at this stage [8], and Gata.a protein was detected in nuclei of endodermal cells. (B) Tcf7 mRNA is expressed strongly in two cells of the brain [8], and Tcf7 protein was detected in nuclei of the two cells in the brain. (C) Zic-r.a mRNA is expressed widely in the nervous system [8, 15], and Zic-r.a protein was detected in nuclei of cells in the nervous system. (PDF) [file pgen.1006045.s004.pdf]

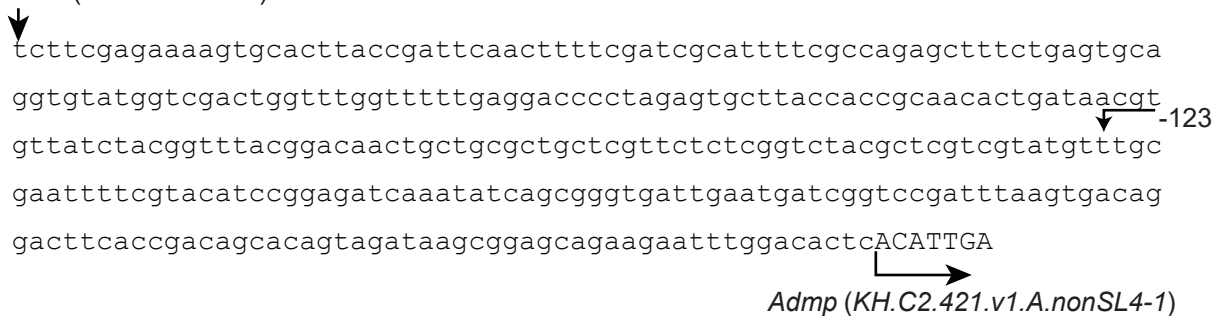

Supplement: S5 Fig — (A) The upstream nucleotide sequence of Tbx6.b sufficient for driving reporter expression specifically in the posterior vegetal cells. Core sequences of the critical Tcf7-binding sites are shown in magenta, and the mutant sequences are shown below each of them in cyan. The positions of mutations shown in Fig 5D are enclosed by black boxes, and the mutant sequences are also shown in cyan. (B) Analysis of a regulatory region of Wnttun5. Illustrations on the left depict the constructs. Green boxes indicate the Gfp reporter gene and SV40 polyadenylation signal. The numbers indicate the relative nucleotide positions from the transcription start site of Wnttun5. Mutant Tcf7-binding sites are indicated by X. Graphs show the percentage of blastomeres expressing the reporter in the anterior vegetal blastomeres, in the posterior vegetal blastomeres, and in the animal blastomeres. (C, D) Images showing expression of the reporter in embryos electroporated with (C) the third and (D) last constructs shown in (B). Scale bar, 100 μm. (E) The upstream nucleotide sequence of Wnttun5 required for driving reporter expression specifically in the posterior vegetal hemisphere. Core sequences of the critical Tcf7-binding sites are shown in magenta, and the mutantd sequences are shown below each of them in cyan. (F) Gel-shift analysis showing that the distal Tcf7-binding site in the upstream region of Wnttun5 did not bind GST protein but bound the Tcf7-GST fusion protein. The shifted band disappeared by incubation with a specific competitor, but not a competitor with a mutant Tcf7-binding site. (G) Analysis of a regulatory region of Admp. Illustrations on the left depict the constructs. The numbers indicate the relative nucleotide positions from the transcription start site of Admp. Graphs show the percentage of blastomeres expressing the reporter in the anterior vegetal blastomeres, in the posterior vegetal blastomeres, and in the animal blastomeres. (H) The upstream nucleotide sequence of [file pgen.1006045.s006.pdf]

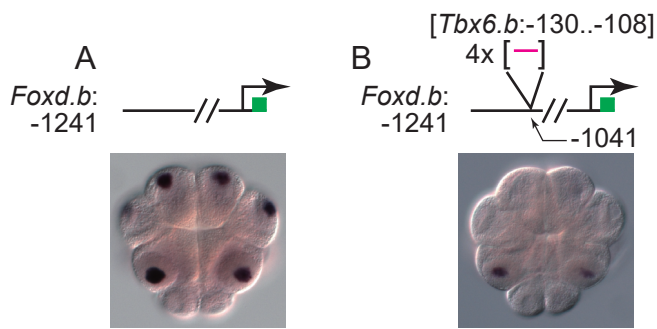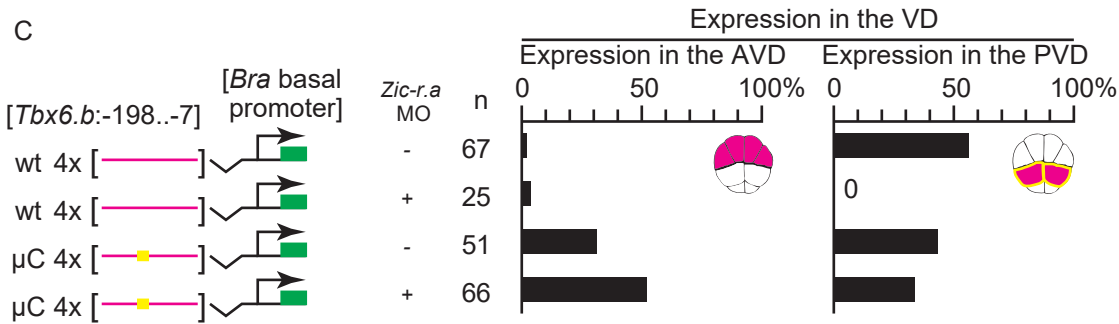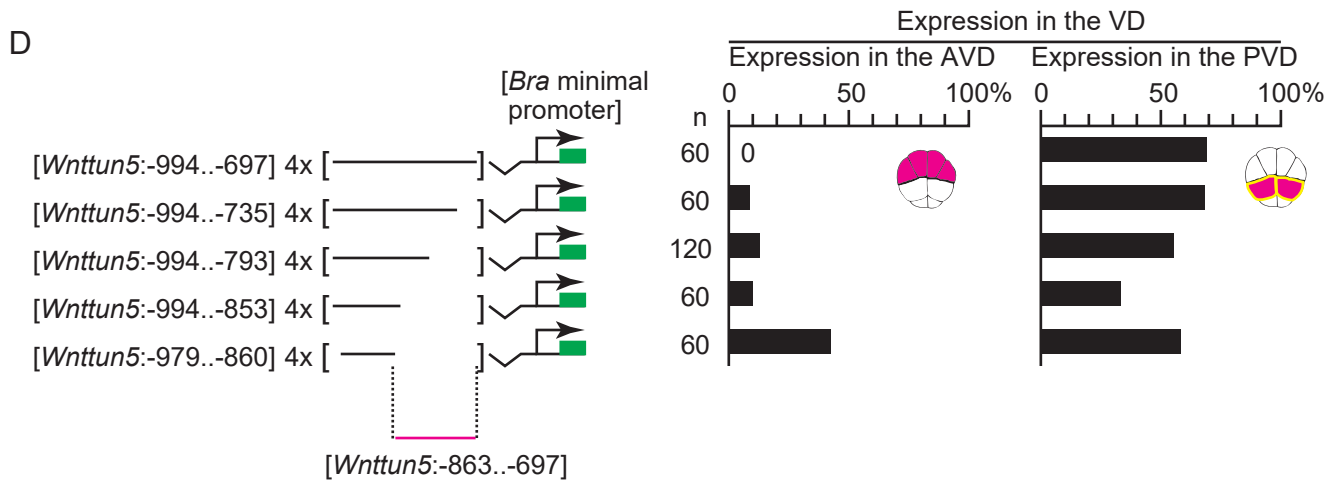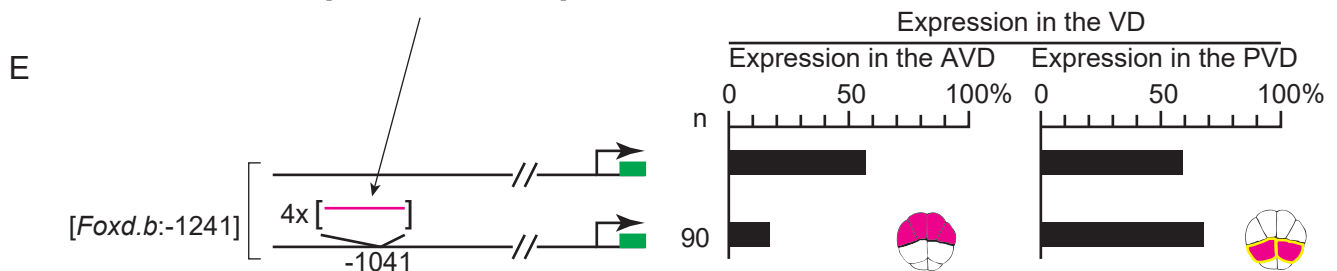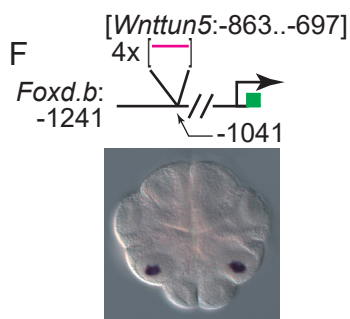

Supplement: S6 Fig — (A)While the reporter gene was expressed in the anterior and posterior vegetal blastomeres under the control of the 1241 bp upstream sequence of Foxd.b, (B) insertion of four repeats of the 22 bp sequence within the upstream region of Tbx6.b suppressed the expression in the anterior vegetal cells. Images are embryos expressing the third and fourth constructs shown in Fig 5G. (C) The repressive element of Tbx6.b directed specific expression in the posterior vegetal cells in a manner dependent on Zic-r.a activity. Constructs depicted in the illustrations on the left were injected with or without an MO against Zic-r.a. The green boxes indicate the Gfp reporter gene and SV40 polyadenylation signal. Graphs on the right show the percentage of blastomeres expressing the reporter gene in the anterior vegetal blastomeres and in the posterior vegetal blastomeres. (D) A series of deletion constructs using the Brachyury basal promoter revealed a repressive element in the upstream sequence of Wnttun5. Illustrations on the left depict the constructs. Graphs show the percentage of blastomeres expressing the reporter in the anterior vegetal blastomeres, and in the posterior vegetal blastomeres. (E) The repressive element, which was identified in (D), was inserted into −1041 of the upstream sequence of Foxd.b. The graphs indicate that this insertion made the expression of the reporter specific for the posterior vegetal cells. Because no expression in the animal hemisphere was observed with the constructs shown in (C), (D) and (E), graphs for expression in the animal hemisphere are omitted. (F) Image showing expression of the reporter with the second construct shown in (E). (PDF) [file pgen.1006045.s007.pdf]

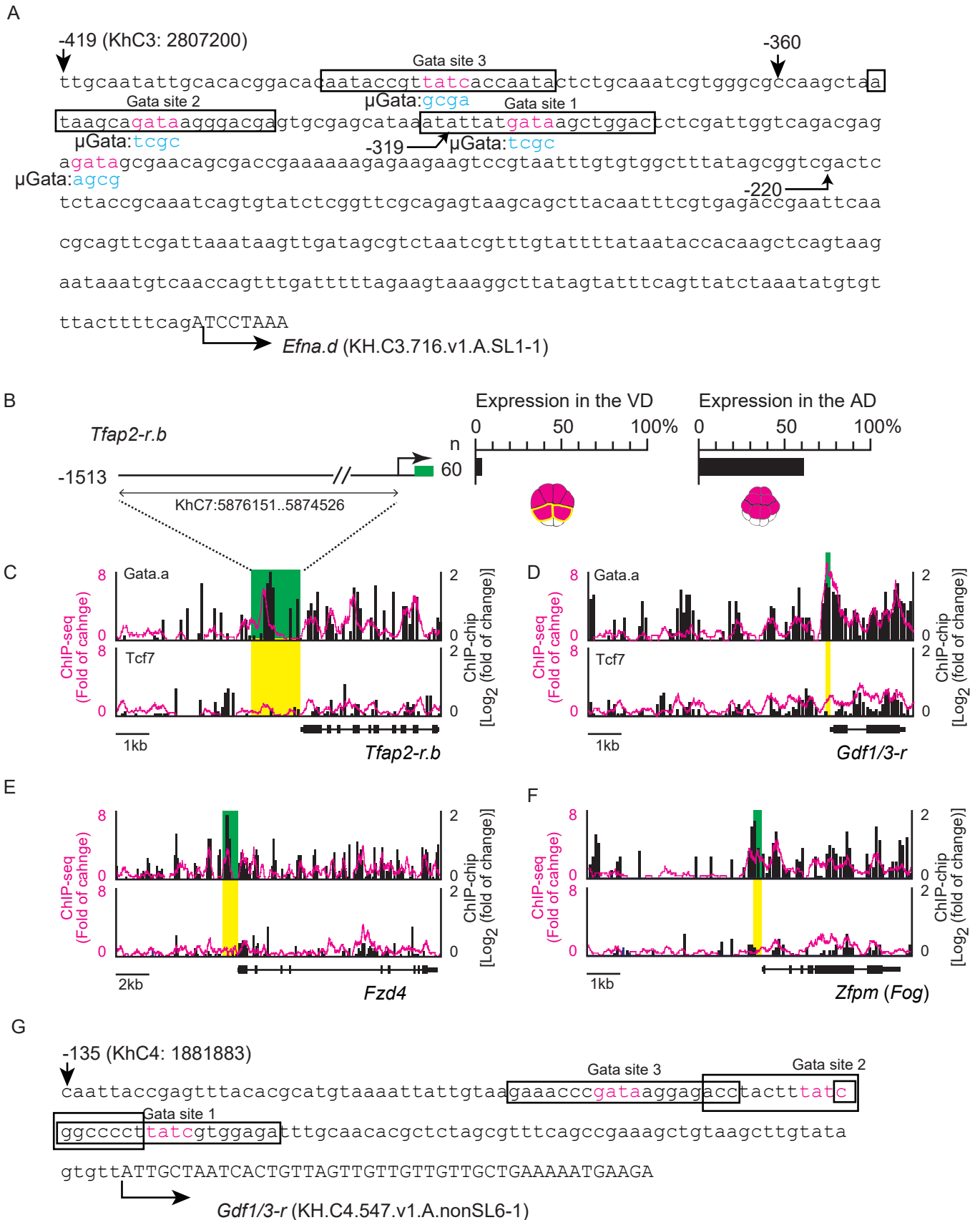

Supplement: S7 Fig — (A) The upstream nucleotide sequence of Efna.d sufficient for driving reporter expression specifically in the animal hemisphere. Core sequences of the critical Gata-binding sites are shown in magenta, and the mutant sequences (Fig 6A) are shown below each of them in cyan. Nucleotide sequences enclosed by boxes were used for the gel-shift assays shown in Fig 7. (B) The 1513 bp upstream sequence of Tfap2-r.b drove reporter expression specifically in the animal hemisphere. The illustration on the left depicts the construct. The green boxes indicate the Gfp reporter gene and SV40 polyadenylation signal. Graphs show the percentage of blastomeres expressing the reporter in the anterior and posterior vegetal blastomeres, and in the animal blastomeres. (C–F) Mapping of the Gata.a and Tcf7 ChIP data onto genomic regions consisting of the exons and upstream regions of (C) Tfap2-r.b, (D) Gdf1/3-r, (E) Fzd4, and (F) Zfpm. The ChIP-chip data are shown in bars and the ChIP-seq data are shown as magenta lines. Each graph shows the fold enrichment (y-axis) for the chromosomal regions (x-axis). Green and yellow boxes indicate the regions sufficient for specific expression that were revealed by the reporter gene assays shown in (B) and previous studies [13, 27]. Regions indicated by green boxes overlap peak regions identified by the peak caller programs for ChIP-seq and ChIP-chip, while the peak caller programs did not identify peaks within regions indicated by yellow boxes. (G) The upstream nucleotide sequence of Gdr1/3-r sufficient for driving reporter expression specifically in the animal hemisphere. Core sequences of the critical Gata-binding sites are shown in magenta. Nucleotide sequences enclosed by boxes were used for gel-shift assays shown in Fig 7. (PDF) [file pgen.1006045.s008.pdf]

A

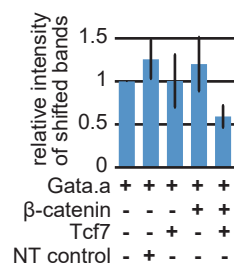

B

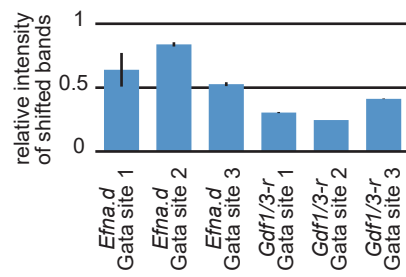

Supplement: S8 Fig — (A) Shifted bands in the gel-shift assay shown in Fig 7B were quantified, and the relative intensity of shifted bands against the band in lane 2 of Fig 7B is shown. (B) Shifted bands in the gel-shift assay shown in Fig 7C were quantified, and the relative intensity of shifted bands is shown. Note that the intensity of shifted bands in lanes with only Gata.a protein may be underestimated as in this case probes were not used in sufficient excess. Black lines indicate standard deviations of two independent experiments. (PDF) [file pgen.1006045.s009.pdf]

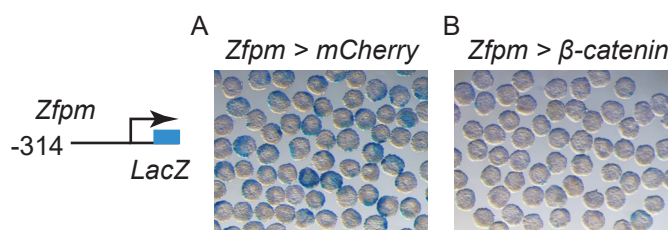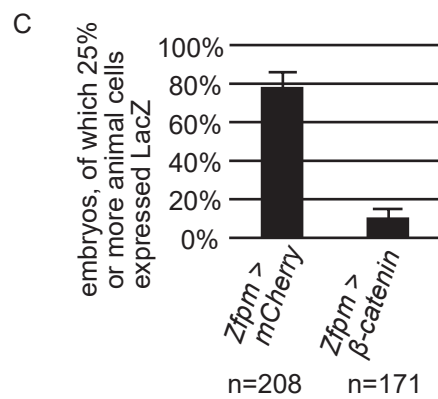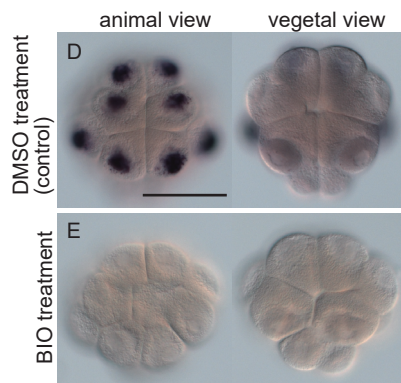

Supplement: S9 Fig — (A–C) A LacZ reporter construct containing the 314 bp upstream sequence of Zfpm was introduced by electroporation together with (A) an mCherry overexpression construct and (B) β-catenin overexpression construct. LacZ activity was detected at the gastrula stage using X-gal. (C) Stained embryos, in which 25% or more animal cells expressed the reporter, are shown. Error bars indicate standard deviations of two independent experiments. (D, E) Expression of Efna.d was suppressed in embryos incubated in sea water (D) without or (E) with BIO. Scale bar, 100 μm. (PDF) [file pgen.1006045.s010.pdf]

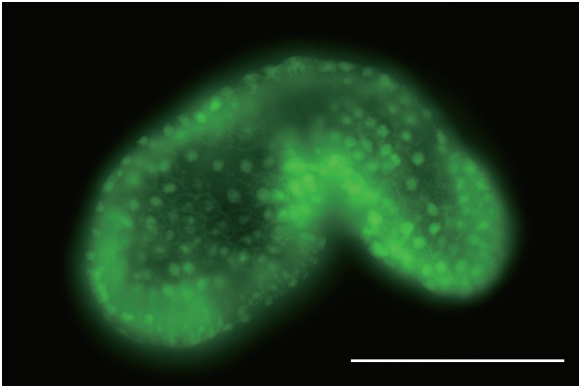

Supplement: S10 Fig — GFP, which was expressed using the Dlx.b upstream sequence, was observed in the nuclei and cytoplasm of a tailbud embryo. Scale bar, 100 μm. (PDF) [file pgen.1006045.s011.pdf]

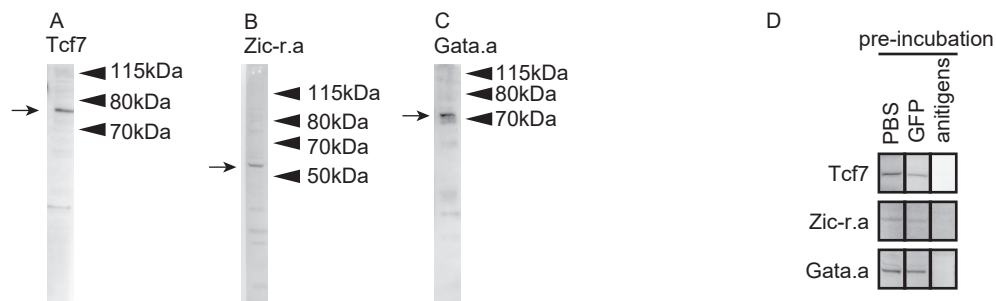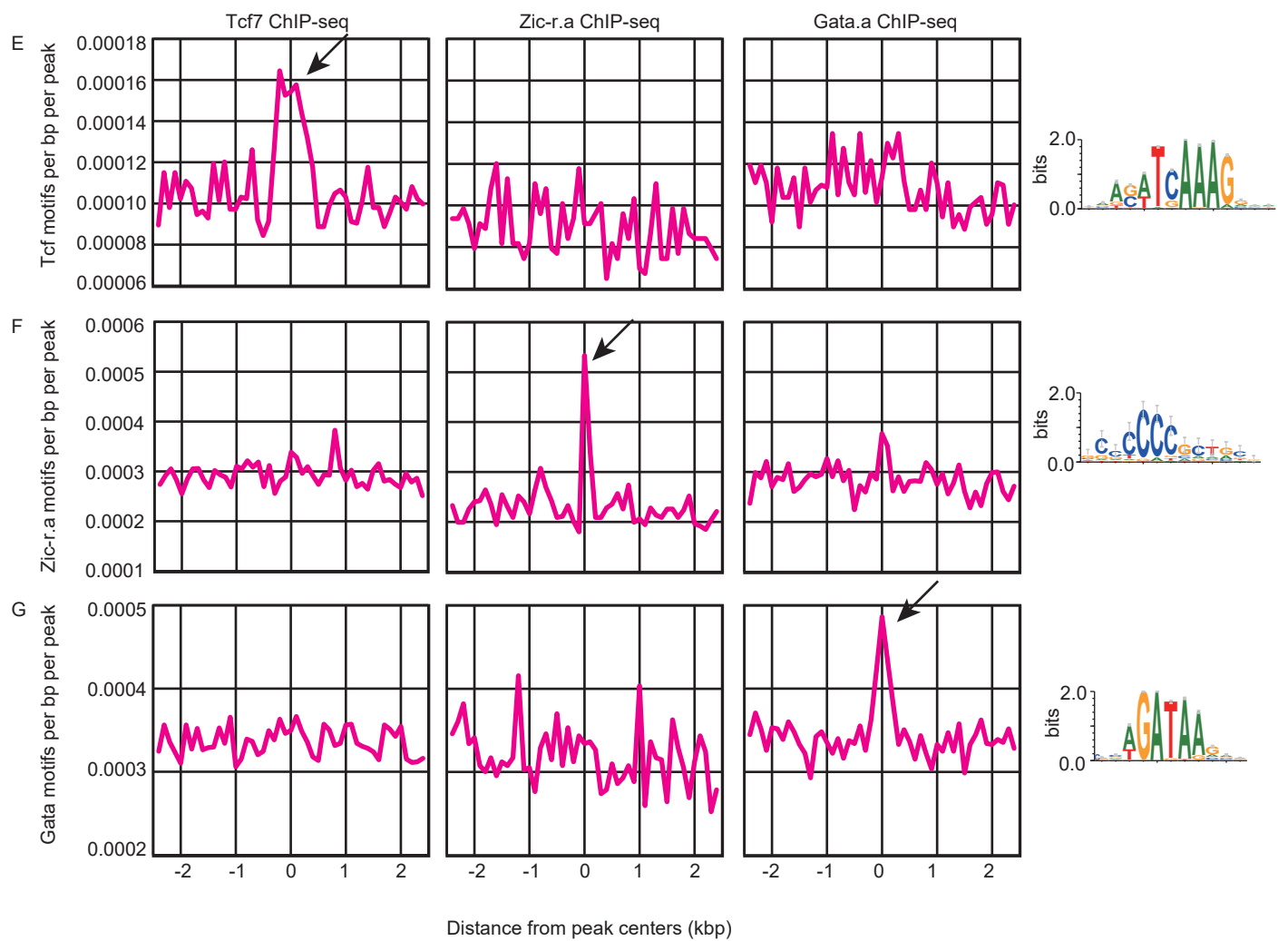

Supplement: S11 Fig — (A–C) Western blot analyses showed that Tcf7, Zic-r.a, and Gata.a were specifically recognized by the antibodies used in the present study. A lysate of unfertilized eggs was used for all experiments. (D) Pre-adsorption tests to confirm antibody specificity. Specific bands recognized by anti-Gata.a, -Tcf7, and -Zic-r.a antibodies were diminished by pre-incubation with their antigens but not with GFP (S11D). (E–G) Enrichment of binding motifs of (E) human TCF7L2, (F) Ciona Zic-r.a and (G) mouse Gata1 shown on the right around peaks identified in ChIP-seq data using antibodies against Tcf7, Zic-r.a, and Gata.a. (PDF) [file pgen.1006045.s012.pdf]

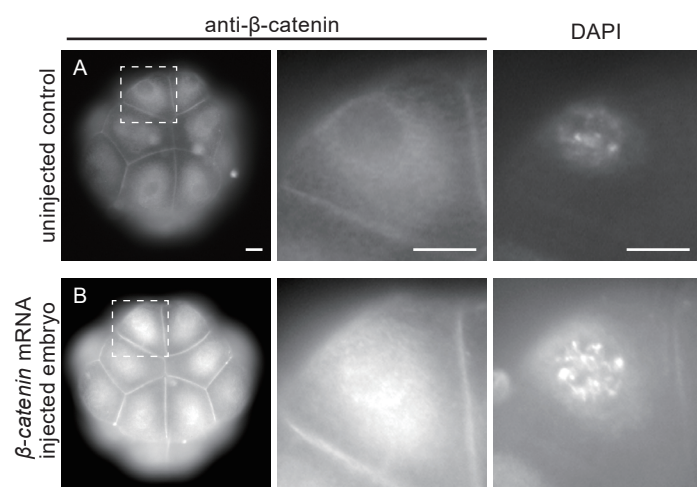

Supplement: S12 Fig — (A) β-catenin was not detected in nuclei of cells in the animal hemisphere of a control embryo, while β-catenin associated with the cell membrane was detected readily. (B) β-catenin was clearly detected in nuclei of cells in the animal hemisphere of an embryo injected with β-catenin mRNA. High magnification views of regions enclosed by dashed-lines in the left panels are shown in the middle panels. Nuclei are indicated in the right panels by DAPI staining. Scale bars, 20 μm. (PDF) [file pgen.1006045.s013.pdf]
