# Supplementary material for: A Maternal System Initiating the Zygotic Developmental Program through Combinatorial Repression in the Ascidian Embryo
Source: PLoS Genet. 2016 May 6;12(5):e1006045. doi: 10.1371/journal.pgen.1006045 (PMC4859511; doi:10.1371/journal.pgen.1006045)
Supplement: S4 Fig — (A) The upstream nucleotide sequence of Fgf9/16/20 sufficient for driving reporter expression specifically in the vegetal hemisphere. Core sequences of the critical Tcf7-binding sites are shown in magenta, and the mutant sequences are shown below each of them in cyan. (B) Analysis of a regulatory region in Foxd.b. Illustrations on the left depict the constructs. Green boxes indicate the Gfp reporter gene and SV40 polyadenylation signal. The numbers indicate the relative nucleotide positions from the transcription start site of Foxd.b. Mutant Tcf7-binding sites are indicated by X. Graphs show the percentage of blastomeres expressing the reporter in the anterior vegetal blastomeres, in the posterior vegetal blastomeres, and in the animal blastomeres. (C) The upstream nucleotide sequence of Foxd.b required for driving reporter expression specifically in the anterior and posterior vegetal hemisphere. Core sequences of the critical Tcf7-binding sites are shown in magenta, and the mutant sequences are shown below each of them in cyan. (D) Alignment of the Foxd.b upstream sequence with the upstream sequence of its paralog, Foxd.a. (E) Gel-shift analysis showing that the proximal Tcf7 binding site in the upstream region of Foxd.b did not bind GST protein but bound the Tcf7-GST fusion protein. The shifted band disappeared by incubation with a specific competitor, but not a competitor with a mutant Tcf7-binding site. (F) The 769 bp upstream sequence of Lefty was sufficient for specific expression in the vegetal hemisphere. (G) Mapping of the Tcf7 ChIP data onto a genomic region consisting of the exons and upstream region of Lefty. The ChIP-chip data are shown in bars and the ChIP-seq data are shown as a magenta line. Each graph shows the fold enrichment (y-axis) for the chromosomal regions (x-axis). A green box indicates the essential upstream sequence of Lefty shown in (F). This region overlapped peaks identified by the peak caller programs for ChIP-seq and ChIP-chip. Nucleo [file pgen.1006045.s005.pdf]

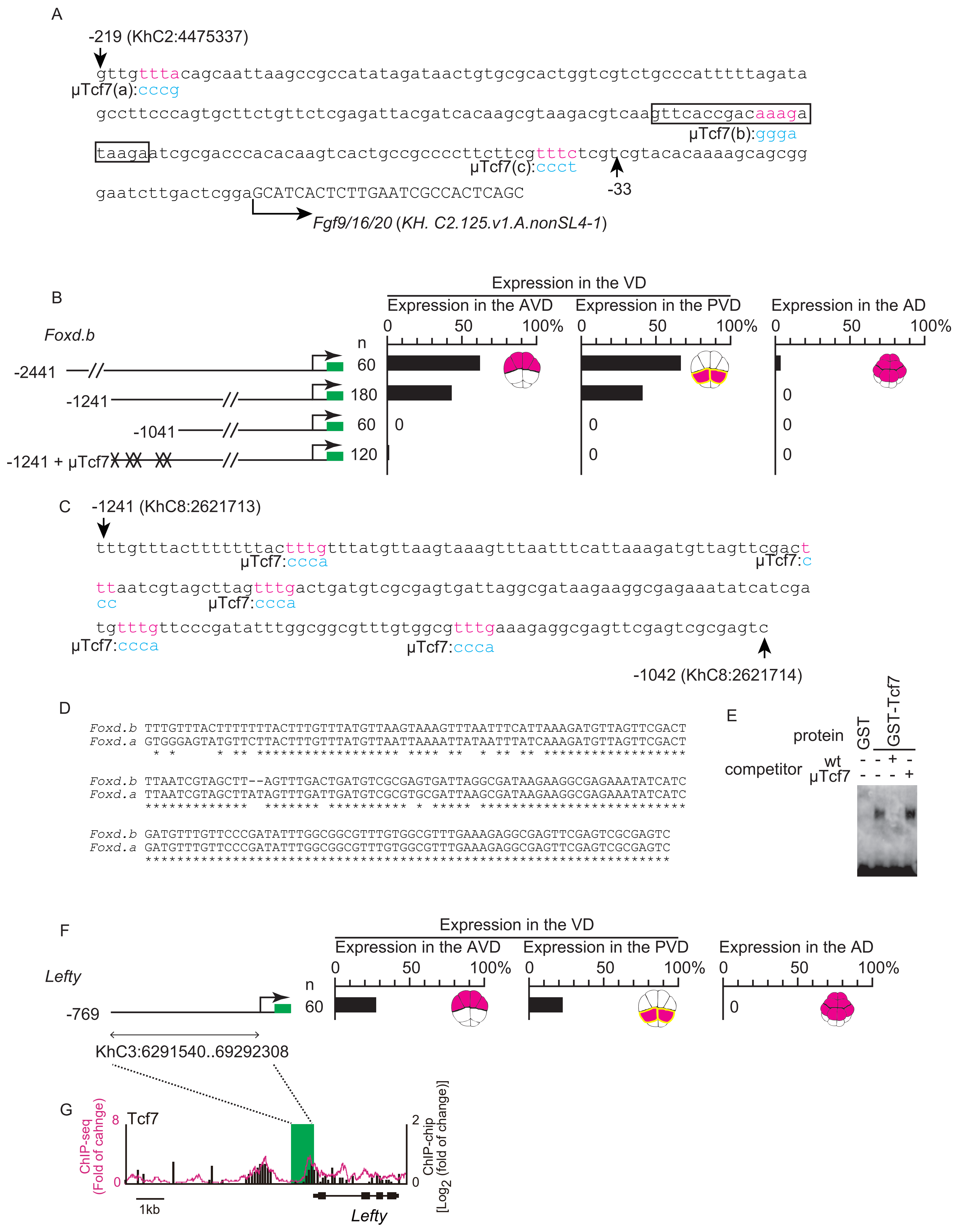
A
-219 (KhC2:4475337)
gttgtttacagcaattaagccgccatatagataactgtgcgcactggtcgtctgcccatttttagata
μTcf7(a):cccg
gccttcccagtgcttctgttctcgagattacgatcacaagcgtaagacgtcaagttcaccgacaaaga
μTcf7(b):ggga
taagaatcgcgacccacacaagtcactgccgccccttcttcgtttctcgtcgtacacaaaagcagcgg
μTcf7(c):ccct
-33
gaatcttgactcggaGCATCACTCTTGAATCGCCACTCAGC
Fgf9/16/20 (KH. C2.125.v1.A.nonSL4-1)
B
Foxd.b
-2441
-1241
-1041
-1241 + μTcf7
n
60
180
60
120
Expression in the VD
Expression in the AVD
Expression in the PVD
Expression in the AD
0
50
100%
C
-1241 (KhC8:2621713)
tttgtttactttttttactttgtttatgttaagtaaagtttaatttcattaaagatgttagttcgact
μTcf7:ccca
μTcf7:c
ttaatcgtagcttagtttgactgatgtcgcgagtgattaggcgataagaaggcgagaaatatcatcga
cc
μTcf7:ccca
tgtttgttcccgatatttggcggcgtttgtggcgtttgaaagaggcgagttcgagtcgcgagtc
μTcf7:ccca
μTcf7:ccca
-1042 (KhC8:2621714)
D
Foxd.b TTTGTTTACTTTTTTTACTTTGTTTATGTTAAGTAAAGTTTAATTTCATTAAAGATGTTAGTTCGACT
Foxd.a GTGGGAGTATGTTCTTACTTTGTTTATGTTAATTAAAATTATAATTTATCAAAGATGTTAGTTCGACT
Foxd.b TTAATCGTAGCTT--AGTTTGACTGATGTCGCGAGTGATTAGGCGATAAGAAGGCGAGAAATATCATC
Foxd.a TTAATCGTAGCTTATAGTTTGATTGATGTCGCGTGCGATTAAGCGATAAGAAGGCGAGAAATATCATC
Foxd.b GATGTTTGTTCCCGATATTTGGCGGCGTTTGTGGCGTTTGAAAGAGGCGAGTTCGAGTCGCGAGTC
Foxd.a GATGTTTGTTCCCGATATTTGGCGGCGTTTGTGGCGTTTGAAAGAGGCGAGTTCGAGTCGCGAGTC
E
protein
GST
GST-Tcf7
competitor
wt
μTcf7
F
Lefty
-769
n
60
KhC3:6291540..69292308
Expression in the VD
Expression in the AVD
Expression in the PVD
Expression in the AD
G
Tcf7
ChIP-seq (Fold of cahnge)
ChIP-chip [Log2 (fold of change)]
1kb
Lefty
