# Supplementary figures and images for: Correction: A Maternal System Initiating the Zygotic Developmental Program through Combinatorial Repression in the Ascidian Embryo
Source: PLoS Genet. 2016 Oct 14;12(10):e1006392. doi: 10.1371/journal.pgen.1006392 (PMC5065148; doi:10.1371/journal.pgen.1006392)

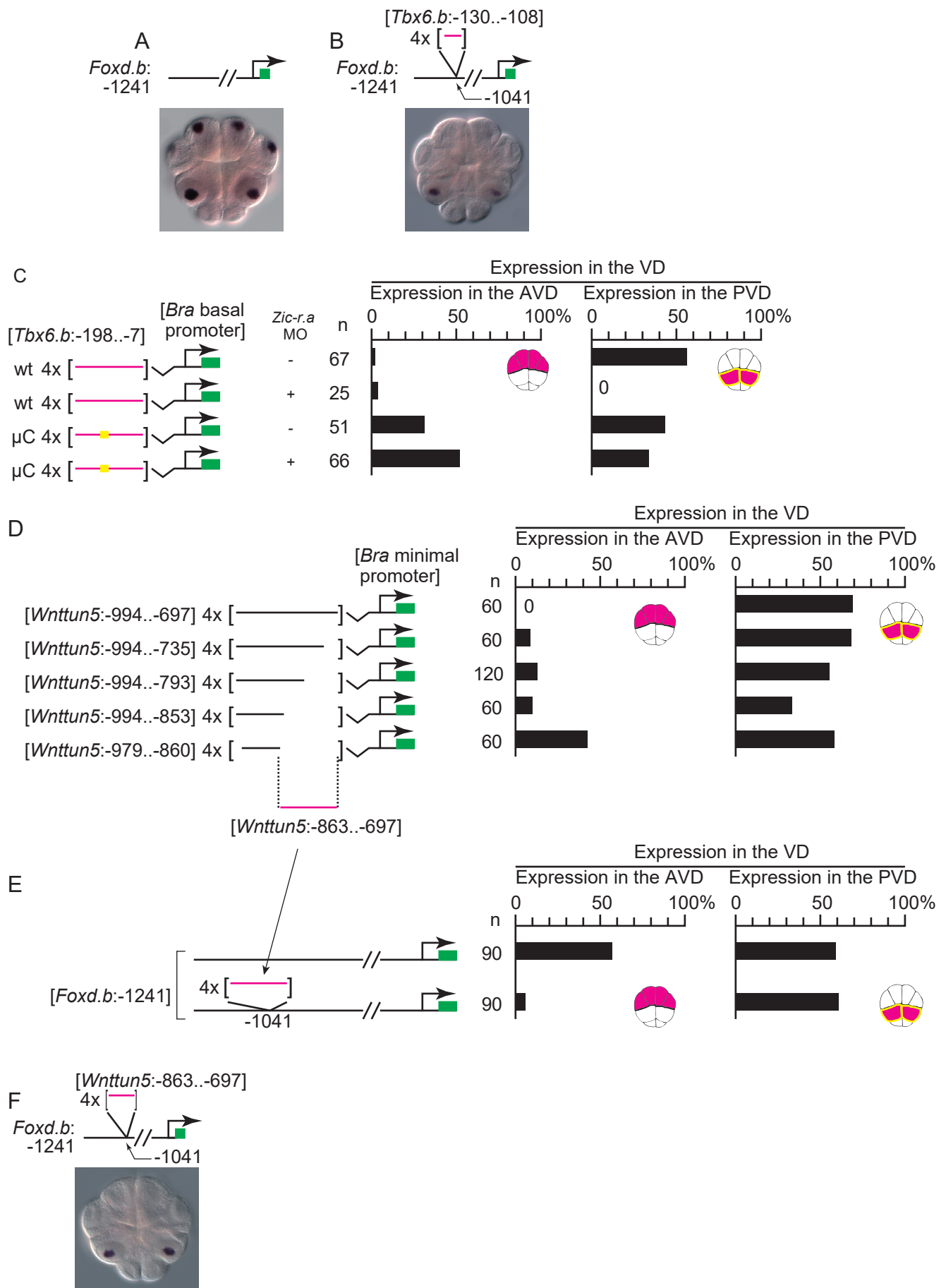

Supplement: S6 Fig — (A) While the reporter gene was expressed in the anterior and posterior vegetal blastomeres under the control of the 1241 bp upstream sequence of Foxd.b, (B) insertion of four repeats of the 22 bp sequence within the upstream region of Tbx6.b suppressed the expression in the anterior vegetal cells. Images are embryos expressing the third and fourth constructs shown in Fig 5G. (C) The repressive element of Tbx6.b directed specific expression in the posterior vegetal cells in a manner dependent on Zic-r.a activity. Constructs depicted in the illustrations on the left were injected with or without an MO against Zic-r.a. The green boxes indicate the Gfp reporter gene and SV40 polyadenylation signal. Graphs on the right show the percentage of blastomeres expressing the reporter gene in the anterior vegetal blastomeres and in the posterior vegetal blastomeres. (D) A series of deletion constructs using the Brachyury basal promoter revealed a repressive element in the upstream sequence of Wnttun5. Illustrations on the left depict the constructs. Graphs show the percentage of blastomeres expressing the reporter in the anterior vegetal blastomeres, and in the posterior vegetal blastomeres. (E) The repressive element, which was identified in (D), was inserted into −1041 of the upstream sequence of Foxd.b. The graphs indicate that this insertion made the expression of the reporter specific for the posterior vegetal cells. Because no expression in the animal hemisphere was observed with the constructs shown in (C), (D) and (E), graphs for expression in the animal hemisphere are omitted. (F) Image showing expression of the reporter with the second construct shown in (E). (PDF) [file pgen.1006392.s001.pdf]
